# The Chinese herb Styrax triggers pharmacokinetic herb-drug interactions *via* inhibiting intestinal CYP3A

**DOI:** 10.3389/fphar.2022.974578

**Published:** 2022-08-29

**Authors:** Feng Zhang, Tiantian Zhang, Jiahao Gong, Qinqin Fang, Shenglan Qi, Mengting Li, Yan Han, Wei Liu, Guangbo Ge

**Affiliations:** ^1^ Department of Neurology, Yueyang Hospital of Integrated Traditional Chinese and Western Medicine, Shanghai University of Traditional Chinese Medicine, Shanghai, China; ^2^ Shanghai Frontiers Science Center of TCM Chemical Biology, Institute of Interdisciplinary Integrative Medicine Research, Shanghai University of Traditional Chinese Medicine, Shanghai, China; ^3^ School of Pharmacy, Zunyi Medical University, Zunyi, China; ^4^ Key Laboratory of Liver and Kidney Diseases (Ministry of Education), Institute of Liver Diseases, Shanghai Key Laboratory of Traditional Chinese Clinical Medicine, Shuguang Hospital Affiliated to Shanghai University of Traditional Chinese Medicine, Shanghai, China

**Keywords:** herb-drug interactions (HDIs), styrax, pentacyclic triterpenoid acids (PTAs), intestinal CYP3A, CYP3A-substrate drugs

## Abstract

Human cytochrome P450 3A4 (hCYP3A4) is a predominant enzyme to trigger clinically relevant drug/herb-drug interactions (DDIs or HDIs). Although a number of herbal medicines have been found with strong anti-hCYP3A4 effects *in vitro*, the *in vivo* modulatory effects of herbal medicines on hCYP3A4 and their potential risks to trigger HDIs are rarely investigated. Herein, we demonstrate a case study to efficiently find the herbal medicine(s) with potent hCYP3A4 inhibition *in vitro* and to accurately assess the potential HDIs risk *in vivo.* Following screening over 100 herbal medicines, the Chinese herb Styrax was found with the most potent hCYP3A4 inhibition in HLMs. *In vitro* assays demonstrated that Styrax could potently inhibit mammalian CYP3A in liver and intestinal microsomes from both humans and rats. *In vivo* pharmacokinetic assays showed that Styrax (i.g., 100 mg/kg) significantly elevated the plasma exposure of two CYP3A-substrate drugs (midazolam and felodipine) when midazolam or felodipine was administered orally. By contrast, the plasma exposure of either midazolam or felodipine was hardly affected by Styrax (i.g.) when the victim drug was administered intravenously. Further investigations demonstrated that seven pentacyclic triterpenoid acids (PTAs) in Styrax were key substances responsible for CYP3A inhibition, while these PTAs could be exposed to intestinal tract at relatively high exposure levels but their exposure levels in rat plasma and liver were extremely low. These findings well explained why Styrax (i.g.) could elevate the plasma exposure of victim drugs only when these agents were orally administrated. Collectively, our findings demonstrate that Styrax can modulate the pharmacokinetic behavior of CYP3A-substrate drugs *via* inhibiting intestinal CYP3A, which is very helpful for the clinical pharmacologists to better assess the HDIs triggered by Styrax or Styrax-related herbal products.

## Introduction

The concomitant use of herbal medicines and Western medicines is widely used in clinical settings for curing various diseases including digestive diseases, cardiovascular and cerebrovascular diseases, infectious diseases, especially in China and other Asia countries (Zhang and Xu, 2012; Su and Geng, 2016; Xu et al., 2017; Qiu et al., 2020). However, the herb-drug interactions (HDIs) caused by the concomitant use of herbal medicines and Western medicines cannot be ignored (Gouws et al., 2012; Fasinu et al., 2015; Gallo et al., 2019; Parvez and Rishi, 2019). A number of studies have shown that cytochrome P450 3A4 (CYP3A4, mainly expressed in liver and small intestine, Supplementary Figure S1, https://www.proteinatlas.org/search/CYP3A4) plays a crucial role in the metabolism and detoxification of a wide range of Western medicines *in vivo*, thus acts as a key mediator in herb-drug interactions (HDIs) and drug-drug interactions (DDIs) (Galetin et al., 2007; Zhou, 2008; Tian and Hu, 2014). It has been reported that a wide range of herbal medicines can regulate the pharmacokinetic behavior of some important CYP3A-substrate drugs (especially for those drugs with very narrow therapeutic windows) by inhibiting or inactivating CYP3A, which may trigger clinically significant HDIs and adverse drug reactions (Zhou et al., 2019; Zhang F. et al., 2021; Zhang F. et al., 2022). Therefore, it is urgent and necessary to accurately assess the inhibitory effects of herbal medicines against human CYP3A, and to precisely evaluate the potential risks of herbal medicines to trigger clinically relevant HDIs when they are concomitantly used with CYP3A-substrate drugs.

**FIGURE 1:**
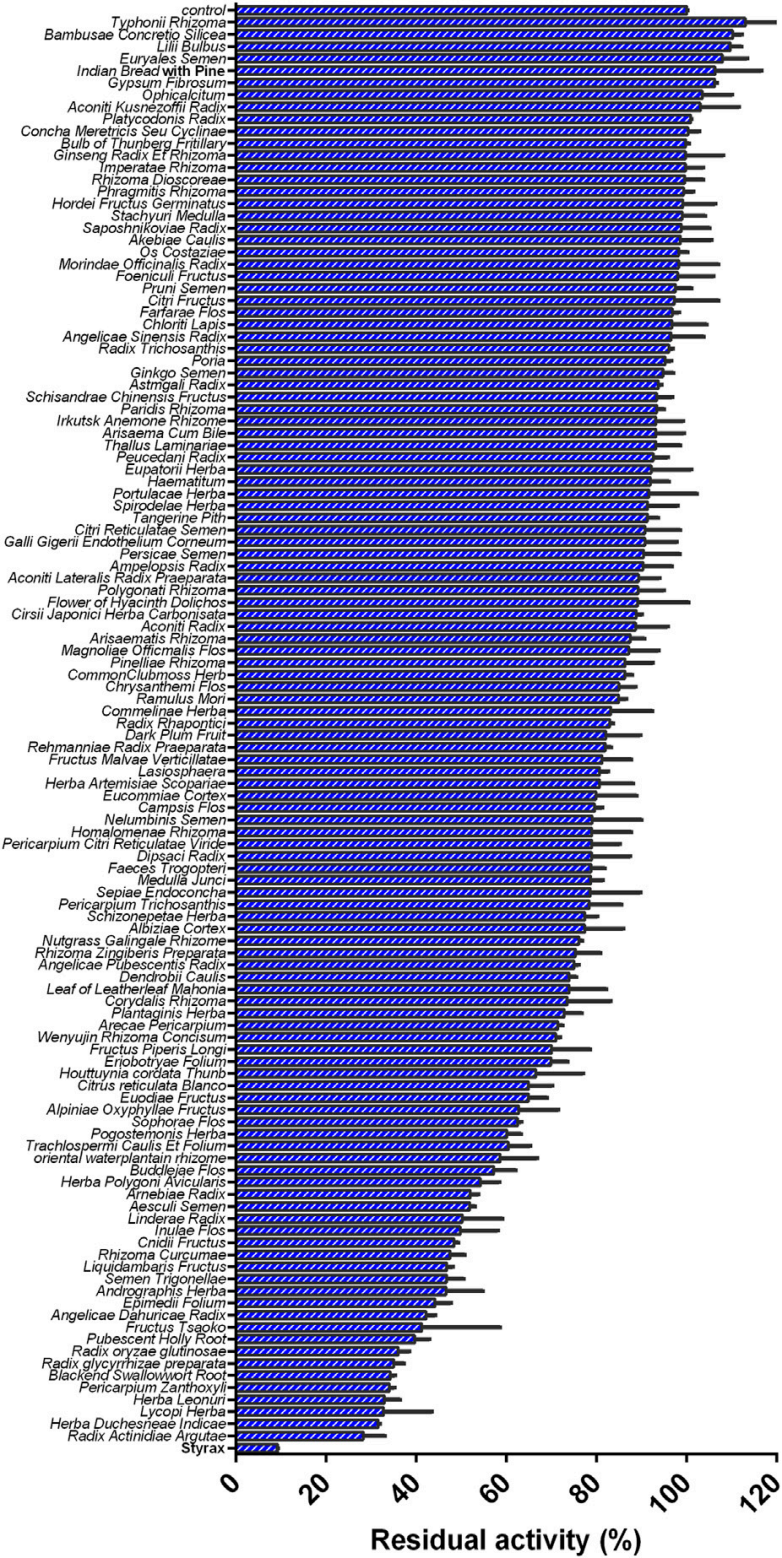
Inhibition effects of 119 Chinese medicine granules/Chinese medicines (100 μg/ml, final concentration) against CYP3A4-catalyzed NEN 4-hydroxylation in HLMs.

Recently, our group and colleagues developed a novel fluorescence-based high-throughput assay for assessing the inhibitory effects of compounds/herbal extracts on human CYP3A4, which offers a powerful platform for investigating CYP3A4-related HDIs or DDIs (Ning et al., 2019; Wu et al., 2021; Jin et al., 2022; Tu et al., 2022). With the help of fluorescence-based high-throughput CYP3A4 inhibition assays, the inhibitory effects of more than 100 herbal medicines against hCYP3A4 were assessed in human liver preparations. The results clearly showed that the Chinese herb Styrax (a herbal medicine used for treating cardiovascular diseases) displayed the most potent inhibitory effect against hCYP3A4 (Figure 1). Notably, Styrax is often used as a key material for preparing some marketed herbal products (such as Suhexiang Pill, Shexiang Baoxin Pill, Subing Dropping Pill, Guanxin Suhe Pill, Shixiang fansheng Pill), owing to the high safety profiles and beneficial effects of this herb in preventing and treating cardiovascular diseases (Bian and Zhang, 2016; Guo et al., 2021). In Asia countries, Styrax and Styrax-related herbal products are frequently used in combination with a panel of marketed cardiovascular drugs (such as warfarin, digoxin and simvastatin) to treat cardiovascular diseases (Zhu, 2009; Tao et al., 2016; Yang et al., 2021). As an important ester spice, Styrax is also used for making a variety of foods (Yu, 2021). Since Styrax is widely used as a key material for making Chinese medicines or foods, the HDIs between this herb and co-administrated Western drugs should be carefully investigated.

The major constituents in Styrax have been reported, in which cinnamic acids, phenylpropanoids and pentacyclic triterpenoid acids (PTAs) are major constituents in this herb, while the PTAs in Styrax are reported as naturally occurring CYP3A inhibitors (Zhang et al., 2020). However, the oral bioavailabilities and plasma exposure of PTAs are extremely low (Wang et al., 2020; Ban et al., 2021; Zhang Y. W. et al., 2021; Ren et al., 2021), and it is not clear whether oral administration of Styrax can inhibit intestinal CYP3A or hepatic CYP3A in living systems and then modulate the pharmacokinetic behavior of CYP3A-substrate drugs *in vivo*. Therefore, this study aims to solve two key questions: 1) Can Styrax change the pharmacokinetic behavior of co-administrated CYP3A-substrate drugs and trigger HDIs *in vivo*; 2) Styrax triggers clinically relevant HDIs *via* inhibiting either hepatic CYP3A4 or intestinal CYP3A4 or both.

To better solve these two questions, two CYP3A-substrate drugs (midazolam and felodipine) were selected as victim drugs in this work, while two administrated ways (oral and intravenous) were used to study the pharmacokinetic interactions between Styrax and two CYP3A-substrate drugs (midazolam and felodipine). Briefly, the inhibitory effects of Styrax against CYP3A-catalyzed midazolam 1′-hydroxylation were carefully assessed in liver and intestinal microsomes from both humans and rats. After then, the pharmacokinetic interactions between Styrax (oral) and CYP3A-substrate drugs (midazolam and felodipine) by two administrated ways (oral and intravenous) were assessed in rats. Furthermore, the inhibitory effects of the PTAs in Styrax on CYP3A were assessed, while the tissue distributions of seven major PTAs in Styrax (the key substances responsible for CYP3A inhibition) were investigated carefully.

## Materials and methods

### Chemicals and reagents

The standard extract of Styrax was acquired by Shanghai Hutchison Pharmaceuticals (Shanghai, China). More than 100 herbal medicines granules were provided by Jiangyin Tianjiang Pharmaceutical Co., Ltd. (Wuxi, Jiangsu, China). Lansoprazole was provided by Hairong (Dujiangyan, Sichuan, China). D-glucose-6-phosphate (G-6-P), glucose-6-phosphate dehydrogenase (G-6-PDH) and β-NADP^+^ were purchased from Sigma-Aldrich (St. Louis, MO, United States). MgCl_2_, 0.9% NaCl, and sodium carboxymethyl cellulose (CMC-Na) were provided by Sinopharm Chemical Reagent (Shanghai, China). The soybean oil was supplied by COFCO Fulinmen Food Marketing Co., Ltd. (China). Midazolam was from J&K Scientific (Shanghai, China). 1′-Hydroxymidazolam was provided by Shanghai Macklin Biochemical Co., Ltd. (Shanghai, China). Felodipine was purchased from Shanghai Yuanye Biotechnology Co., Ltd. (Shanghai, China). Osalmide was ordered from Meilun Bio. Tech (Dalian, China). N-ethyl-1,8-naphthalimide (NEN) and N-ethyl-4-hydroxyl-1,8-naphthalimide (NEHN) were synthesized by using the previously reported protocol (Ning et al., 2019). Betulinic acid, epibetulinic acid, betulonic acid, oleanonic acid, oleanolic acid, maslinic acid and corosolic acid were supplied by Shanghai Standard Technology Co., Ltd. (Shanghai, China). The pooled HLMs (Lot No. H0610), pooled HIMs (Lot No. H0610.I), pooled RIMs (Lot No. R1000.I) were obtained from XenoTech (United States). The pooled RLMs (Lot No. LM-DS-02M) was ordered from Research Institute for Liver Diseases (RILD, Shanghai, China). BCA Protein Quantification Kit was acquired by Thermo Fisher Scientific Co., Ltd. Ultrapure water purified by Milli-Q^®^ Integral Water Purification System (Millipore, United States) was used throughout. LC grade methanol, acetonitrile and formic acid were supplied by Fisher Scientific Co., Ltd. (Fair Lawn, NJ, United States). Other reagents were of the highest grade commercially available. The purities of all compounds used in this study were greater than 98%.

### CYP3A inhibition assays

#### Inhibition of CYP3A4-catalyzed NEN 4-hydroxylation by herbal medicines or natural constituents

The inhibitory effects of more than 100 herbal medicines against CYP3A4 were assayed by using a previously reported fluorescence-based biochemical assay (Ning et al., 2019; Tu et al., 2022). In brief, PBS (pH 7.4, 100 mM), herbal medicines (100 μg/ml, final concentration)/PTAs/solvent, NEN, G-6-P, G-6-PDH, MgCl_2_, and HLMs were added to the 96-well plate in turn, then the samples were vortexed and pre-incubated for 3 min at 37 °C. The oxidative reactions were initiated by adding β-NADP^+^ to yield a final volume of 200 μl, then the formation rates of NEHN (hydroxylated metabolite of NEN) were continuously monitored for 30 min at 37 °C using a 96-well fluorescence microplate reader (SpectraMax^®^ iD3, Molecular Devices, Austria). The percentage of organic solvent in all incubation is not over 1%. Please refer to the supplementary material (Supplementary Table S1) for detailed experimental conditions.

#### Inhibition of CYP3A-catalyzed midazolam 1′-hydroxylation by Styrax or its constituents

The inhibitory effects of Styrax or its constituents against CYP3A were assayed by using midazolam as the substrate (Lee et al., 2013; Dunkoksung et al., 2019; Fang et al., 2020; Tu et al., 2020). Briefly, PBS (pH 7.4, 100 mM), Styrax/PTAs/solvent, midazolam, G-6-P, G-6-PDH, MgCl_2_, and enzyme sources (HLMs/RLMs/HIMs/RIMs, 0.1 mg/ml, final concentrations) were added to the EP tube in turn and vortexed, then the samples were pre-incubated for 3 min at 37 °C. Next, the reactions were initiated by adding β-NADP^+^ to yield a final volume of 200 μl. Following incubation for 30 min at 37 °C, and 200 μl ice-cold acetonitrile containing internal standard (IS, 90 ng/ml) was subsequently added to quench the oxidative reaction. The mixture was then centrifuged at 20,000 ×g, 4 °C for 20 min, and the supernatant was quantified by LC-MS/MS analysis as described in Supplementary Table S2.

### Pharmacokinetic studies

Sprague-Dawley rats (weighing 200–220 g) were purchased from Shanghai Xipuer-Bikai Experimental Animal Co., Ltd. (Shanghai, China, Permit Number: SCXK (Hu) 2018-0006). The animal experiments were carried out in Experimental Animal Center of Shanghai University of Traditional Chinese Medicine (Approval Number: PZSHUTCM210709016) and conducted in accordance with the State Committee of Science and Technology of China. In brief, forty male rats were housed under controlled environmental conditions (22 ± 2°C; 40%–80% relative humidity; 12 h light/dark cycle) for 1 week with free access to food and water. The rats were fasted overnight before dosing but were free to drink water, and provided food after finishing the study. Styrax was suspended in soybean oil. Oral midazolam/felodipine was suspended in 0.5% CMC-Na, and intravenous midazolam/felodipine was suspended in 0.9% NaCl. Styrax or soybean oil was administered orally at a dose of 100 mg/kg. The oral dose of midazolam is 20 mg/kg and the intravenous dose is 5 mg/kg. The oral dose of felodipine is 10 mg/kg and the intravenous dose is 2 mg/kg. The dose of Styrax in rats was referred to the daily recommended oral dose in humans. The dose of midazolam and felodipine in rats was referred to the doses in published literature (Lundahl et al., 1997; Kotegawa et al., 2002; Li et al., 2010; Surya Sandeep et al., 2014). Forty rats were randomly divided into eight groups.

group 1: Styrax + midazolam (oral);
group 2: soybean oil + midazolam (oral);
group 3: Styrax + midazolam (intravenous);
group 4: soybean oil + midazolam (intravenous);
group 5: Styrax + felodipine (oral);
group 6: soybean oil + felodipine (oral);
group 7: Styrax + felodipine (intravenous);
group 8: soybean oil + felodipine (intravenous);

Styrax (100 mg/kg, experiment group) or soybean oil (100 mg/kg, control group) was administered orally. After 30 min, midazolam/felodipine was administered orally and intravenously. For oral midazolam groups, blood samples were collected at 5, 10, 20, 30, 60 min, 2, 3, 4, 6, 9 h. For intravenous midazolam groups, blood samples were collected at 2, 5, 10, 20, 30, 60 min, 2, 3, 4, 6, 9 h. For oral felodipine groups, blood samples were collected at 10, 30, 60 min, 3, 4, 6, 8, 10, 24 h. For intravenous felodipine groups, blood samples were collected at 2, 5, 10, 15, 30, 45, 60 min, 4, 6, 10 h. Then blood samples were centrifuged at 8,000 rpm, 4 °C for 10 min, the supernatant was stored at −80 °C until analysis (Li et al., 2010; Xue et al., 2011; Kallem et al., 2013; Xiang et al., 2017). 50 μl plasma was mixed with 50 μl ultrapure water and 300 μl acetonitrile (containing 50 ng/ml IS). The mixture was vortexed for 1 min and then centrifuged at 10,000 × g, 4 °C for 10 min. The supernatant (320 μl) was dried by nitrogen and dissolved with 80 μl of 10% acetonitrile. The supernatant (5 μl) was injected for UHPLC-Q-Orbitrap HRMS analysis (Mass spectrometry parameters were described in Supplementary Figure S2,S3 & Supplementary Table S3,S4). Both midazolam and felodipine were quantified within the linear range of their calibration curves. The pharmacokinetic parameters of midazolam, felodipine were fitted by standard noncompartmental analyses using PKSolver.

### Tissue distribution of PTAs of Styrax in rats

#### Animal handling and sampling

Nine Male Sprague-Dawley rats (250–300 g) were purchased from Shanghai Sippe-Bk Lab Animal Co., Ltd. (Shanghai, China) and were fed in Experimental Animal Center of SHUTCM. All animals were housed under controlled environmental conditions (22 ± 2°C; 40%–80% relative humidity; 12 h light/dark cycle) for 1 week with free access to food and water. The rats were fasted overnight before dosing but were free to drink water. Nine rats were randomly divided into three groups (1, 2, and 4 h). Styrax (100 mg/kg) was administered orally, then nine rats were anesthetized by pentobarbital after administrating 1, 2, and 4 h, respectively. Blood samples were collected from the abdominal aorta, and the tissues (including the liver, duodenum, jejunum, ileum and colon) were removed rapidly and placed on ice. After then, the blood samples were centrifuged at 8,000 rpm, 4 °C for 10 min, and the supernatant was stored at −80 °C until analysis.

#### Determination of PTAs in rat tissues

Firstly, each tissue (such as liver, duodenum, jejunum, ileum, colon) was accurately weighed after sucking the water on tissue surface. Each tissue was put into an EP tube and then cut (3-5 times) by a scissor. After then, PBS (pH 7.4) and magnetic beads were added into EP tube, grind the tissue by a tissue grinder. The tissue grinding fluid was centrifuged at 9,000 g, 4 °C for 20 min. 50 μl tissue supernatant was diluted by adding 250 μl LC grade acetonitrile. The protein was precipitated by centrifuging at 20,000 ×g, 4 °C for 30 min and the supernatant was collected to determine the protein concentration by a BCA Protein Quantification Kit. After then, the concentrations of seven PTAs were determined by using UHPLC-Q-Orbitrap HRMS analysis. Mass spectrometry parameters were described in Supplementary Figure S4 & Supplementary Table S5.

### Data analysis

The dose-inhibition curves of Styrax/PTAs against CYP3A were depicted by using different dosages of Styrax/PTAs, while the half maximal inhibition concentration (IC_50_) was determined as the concentration of Styrax/PTAs at the catalytic activity of CYP3A was inhibited by 50% compared to the negative control (solvent only). In this study, all assays were tested in triplicate, while the data were shown as mean ± standard deviation (SD). IC_50_ values were fitted by GraphPad Prism 6.0 (GraphPad Software, Inc., La Jolla, United States). Meanwhile, the following formula was used to calculate the residual activities of CYP3A:

The residual activity (%) = (the Peak Area or Fluorescence value of the metabolite in the presence of inhibitor)/the Peak Area or Fluorescence value of the metabolite in negative control (solvent only) × 100%.

## Results

### Screening of the herbal medicines with potent CYP3A4 inhibition

Firstly, with the help of fluorescence-based high-throughput CYP3A4 inhibition assay, the inhibitory potentials of more than 100 herbal medicines against CYP3A4-catalyzed NEN 4-hydroxylation were assayed in HLMs by using a single dose (100 μg/ml, final concentration). As shown in Figure 1 and Supplementary Table S6, all tested herbal medicines showed different inhibitory effects on CYP3A4-catalyzed NEN 4-hydroxylation in HLMs. Among all the tested herbal medicines, the Chinese herb Styrax was found with the most potent hCYP3A4 inhibition in HLMs. At the dose of 100 μg/ml, Styrax inhibited CYP3A4-catalyzed NEN 4-hydroxylation near completely, with the residual activity of 9.25%. By contrast, other herbal medicines displayed relatively weak inhibitory effects on hCYP3A4 at the same dose. These findings clearly demonstrate that the Chinese herb Styrax displays strong CYP3A4 inhibition potency, which may interact with CYP3A4-substrate drugs to trigger HDIs in living systems.

### Inhibitory effects of Styrax against midazolam 1′-hydroxylation

**FIGURE 2:**
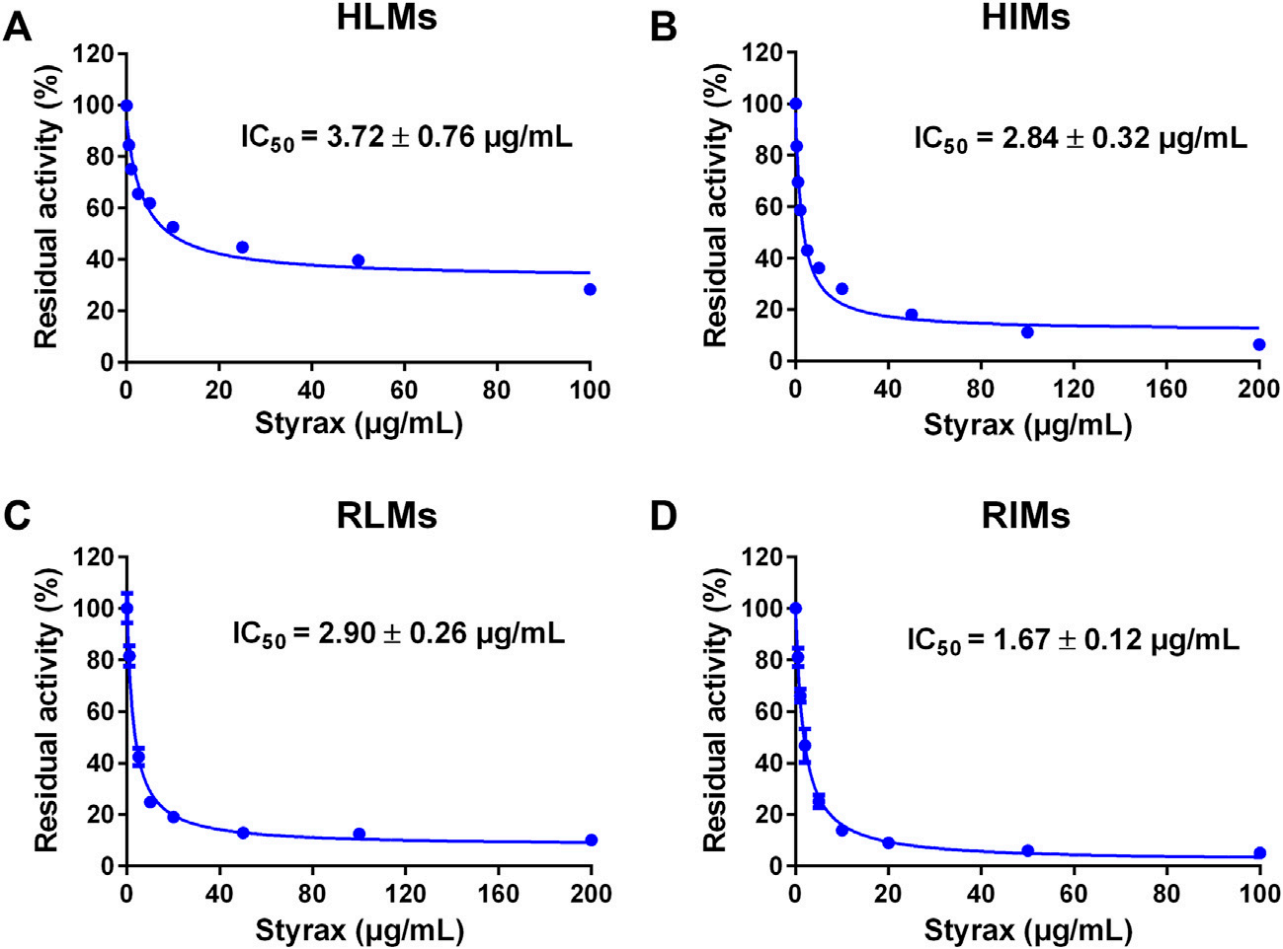
**(A)** Dose-inhibition curve of Styrax extract towards midazolam 1′-hydroxylation in HLMs. **(B)** Dose-inhibition curve of Styrax extract towards midazolam 1′-hydroxylation in HIMs. **(C)** Dose-inhibition curve of Styrax extract towards midazolam 1′-hydroxylation in RLMs. **(D)** Dose-inhibition curve of Styrax extract towards midazolam 1′-hydroxylation in RIMs. Data are expressed as mean ± SD.

Next, the inhibitory effects of Styrax against mammalian CYP3A were validated by using a substrate-drug (midazolam) in different tissue preparations. To this end, the inhibitory effects of Styrax against CYP3A-catalyzed midazolam 1′-hydroxylation were carefully investigated in different enzyme sources including HLMs, HIMs, RLMs, and RIMs. As shown in Figure 2 and Supplementary Table S7, Styrax could potently inhibit CYP3A-catalyzed midazolam 1′-hydroxylation, with IC_50_ values of 3.72 μg/ml, 2.84 μg/ml, 2.90 μg/ml and 1.67 μg/ml, for HLMs, HIMs, RLMs, RIMs, respectively. These findings demonstrated that Styrax could potently inhibit mammalian CYP3A in liver and intestinal microsomes from both human and rat *in vitro*, with similar inhibition tendency and potency. However, it is still unclear that Styrax can inhibit hepatic CYP3A or intestinal CYP3A *in vivo*. Therefore, the *in vivo* pharmacokinetic interactions between Styrax and midazolam were carefully investigated by using two administrated ways (i.v. and i.g.).

### Pharmacokinetic interactions between Styrax and midazolam by i.v. and i.g.

**FIGURE 3:**
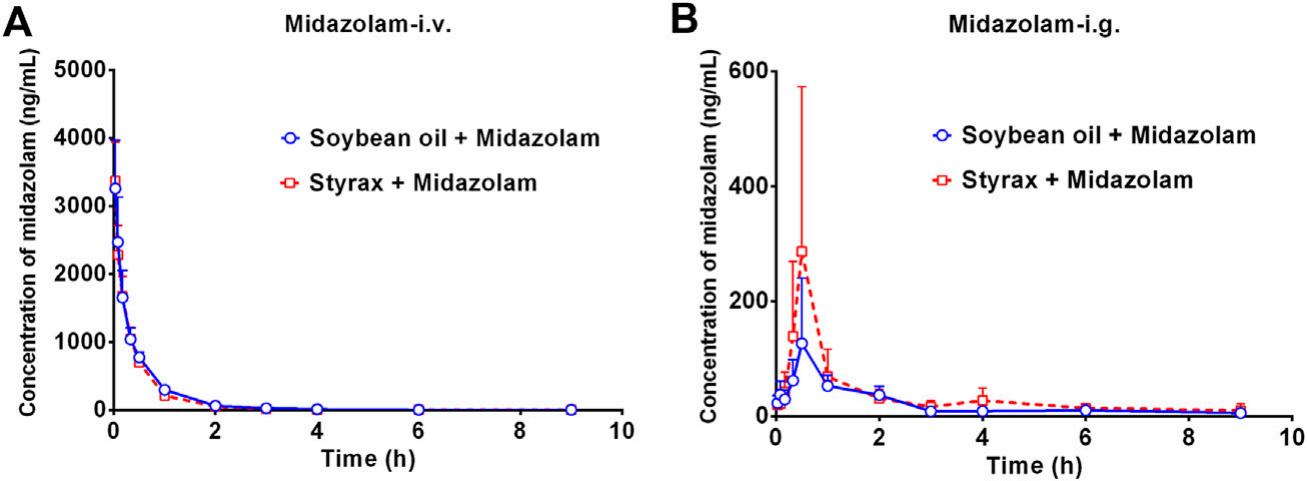
**(A)** The mean plasma concentration-time curves of midazolam after intravenous administration of midazolam (5 mg/kg) in rats pretreated with Soybean oil (blue) or Styrax (i.g. 100 mg/kg, red). **(B)** The mean plasma concentration-time curves of midazolam after intragastric administration of midazolam (20 mg/kg) in rats pretreated with (blue) or Styrax (i.g. 100 mg/kg, red). Data are expressed as the mean ± SD.

Next, the *in vivo* effects of Styrax on the pharmacokinetics of midazolam (i.v. and i.g.) were carefully investigated in rats, by using soybean oil (without Styrax) as control groups. The blood concentration-time profiles of midazolam with two administrated ways (oral and intravenous) were shown in Figure 3 and the pharmacokinetic parameters were detailed in Table 1. Interestingly, the plasma exposure of midazolam was hardly affected by Styrax when midazolam was administered intravenously in combination with Styrax (i.g.) to rats. By contrast, when midazolam (i.g.) was co-administered orally with Styrax (i.g.) to rats, the pharmacokinetic behavior of midazolam was significantly changed compared with the control group (soybean oil instead of Styrax). The AUC_(0-inf)_ of midazolam (i.g.) was increased 2.04-fold (from 211.23 ng/mL·h to 431.20 ng/mL·h), while the C_max_ value of midazolam (i.g.) in rats plasma was increased 2.28-fold (from 129.16 ng/ml to 294.05 ng/ml). Moreover, the metabolic half-life (t_1/2_) of midazolam (i.g.) was prolonged 2.11-fold (from 2.14 to 4.52 h). These findings clearly suggested that Styrax strongly elevated the plasma exposure of midazolam when midazolam was co-administered orally. Considering that both intestinal CYP3A and hepatic CYP3A participate in midazolam metabolism when this agent is administrated orally, while Styrax (i.g.) cannot influence hepatic metabolism of midazolam, suggesting that Styrax may modulate the *in vivo* pharmacokinetic behavior of midazolam (i.g.) *via* inhibiting intestinal CYP3A but not hepatic CYP3A.

**TABLE 1:** Pharmacokinetic parameters of midazolam after administrating midazolam (i.v., 5 mg/kg; i.g., 20 mg/kg) in rats pretreated with Soybean oil, Styrax (100 mg/kg). Data are the mean ± SD.

Route of administration	Group	AUC_(0-inf)_ (ng/mL·h)	C_max_ (ng/ml)	t_1/2_ (h)	T_max_ (h)
Midazolam (i.v.)	Soybean oil + midazolam	1324.24 ± 130.11	3258.43 ± 708.33	0.88 ± 0.28	0.03 ± 0.00
	Styrax + midazolam	1201.37 ± 114.05	3371.29 ± 569.54	0.52 ± 0.14	0.03 ± 0.00
	Ratio	0.91	1.03	0.59	1.00
Midazolam (i.g.)	Soybean oil + midazolam	211.23 ± 87.21	129.16 ± 110.80	2.14 ± 0.28	0.63 ± 0.25
	Styrax + midazolam	431.20 ± 217.14	294.05 ± 277.47	4.52 ± 3.96	0.63 ± 0.25
	Ratio	2.04	2.28	2.11	1.00

### Pharmacokinetic interactions between Styrax and felodipine by i.v. and i.g.

**FIGURE 4:**
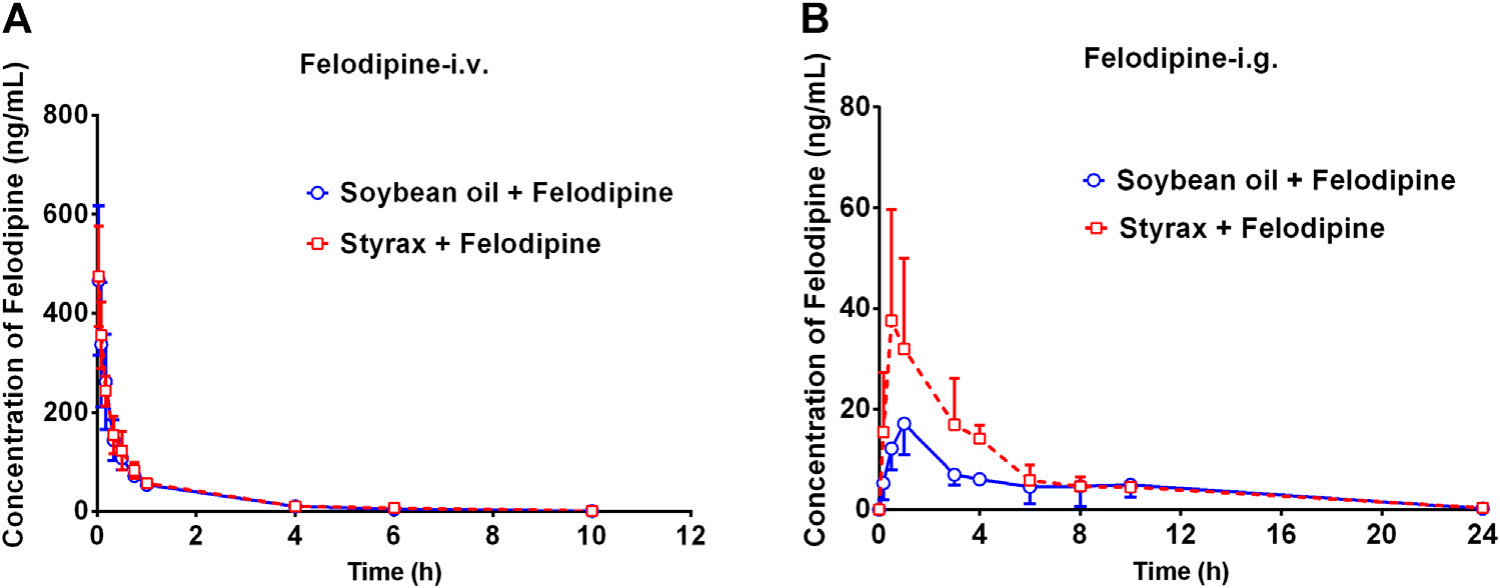
**(A)** The mean plasma concentration-time curves of felodipine after intravenous administration of felodipine (2 mg/kg) in rats pretreated with Soybean oil (blue) or Styrax (i.g. 100 mg/kg, red). **(B)** The mean plasma concentration-time curves of felodipine after intragastric administration of felodipine (10 mg/kg) in rats pretreated with (blue) or Styrax (i.g. 100 mg/kg, red). Data are expressed as the mean ± SD.

To further confirm that Styrax could influence the pharmacokinetic behavior of orally administrated agent, felodipine (an orally administrated CYP3A-substrate drug) was used as a victim drug to assess the pharmacokinetic interactions between Styrax (i.g., 100 mg/kg) and felodipine *via* two administrated ways (i.v. and i.g.). As shown in Figure 4A and Table 2, when felodipine was administered intravenously in rats, Styrax displayed negligible effect on the pharmacokinetic parameters of felodipine. Notably, as shown in Figure 4B and Table 2, compared with the control group (soybean oil instead of Styrax), Styrax significantly increased the values of AUC_(0-inf)_ (1.41-fold, from 111.72 ng/mL·h to 156.98 ng/mL·h) and C_max_ (2.16-fold, from 17.40 ng/ml to 37.58 ng/ml) of felodipine when felodipine was administered orally in combination with Styrax (i.g.) in rats. These findings further suggest that Styrax can modulate the pharmacokinetic behavior of orally administrated CYP3A-substrate drugs (midazolam and felodipine) by inhibiting intestinal CYP3A but not hepatic CYP3A *in vivo*.

**TABLE 2:** Pharmacokinetic parameters of felodipine after administrating felodipine (i.v., 2 mg/kg; i.g., 10 mg/kg) in rats pretreated with Soybean oil, Styrax (100 mg/kg). Data are the mean ± SD.

Route of administration	Group	AUC_(0-inf)_ (ng/mL·h)	C_max_ (ng/ml)	t_1/2_ (h)	T_max_ (h)
Felodipine (i.v.)	Soybean oil + felodipine	274.51 ± 51.21	466.24 ± 151.20	1.41 ± 0.41	0.03 ± 0.00
	Styrax + felodipine	294.99 ± 34.21	474.57 ± 101.10	1.44 ± 0.31	0.03 ± 0.00
	Ratio	1.07	1.02	1.02	1.00
Felodipine (i.g.)	Soybean oil + felodipine	111.72 ± 18.92	17.40 ± 5.86	4.83 ± 3.05	1.30 ± 0.97
	Styrax + felodipine	156.98 ± 44.70	37.58 ± 22.16	3.81 ± 1.99	0.50 ± 0.00
	Ratio	1.41	2.16	0.79	0.38

### Chemical profiling of Styrax

Next, the chemical constituents in Styrax were analyzed by using UHPLC-Q-Exactive Orbitrap HRMS. The total ion chromatograms (TICs) of Styrax were shown in Supplementary Figure S5. A total of 21 chemical constituents were identified in Styrax by comparing the retention times, MS fragmentation behavior, literature information and the MS/MS databases of natural products. The major constituents in Styrax are cinnamic acids, phenylpropanoids and PTAs. The mass spectrometry data of the identified constituents in Styrax were summarized in Supplementary Table S8.

### The inhibitory effects of seven PTAs on CYP3A

**FIGURE 5:**
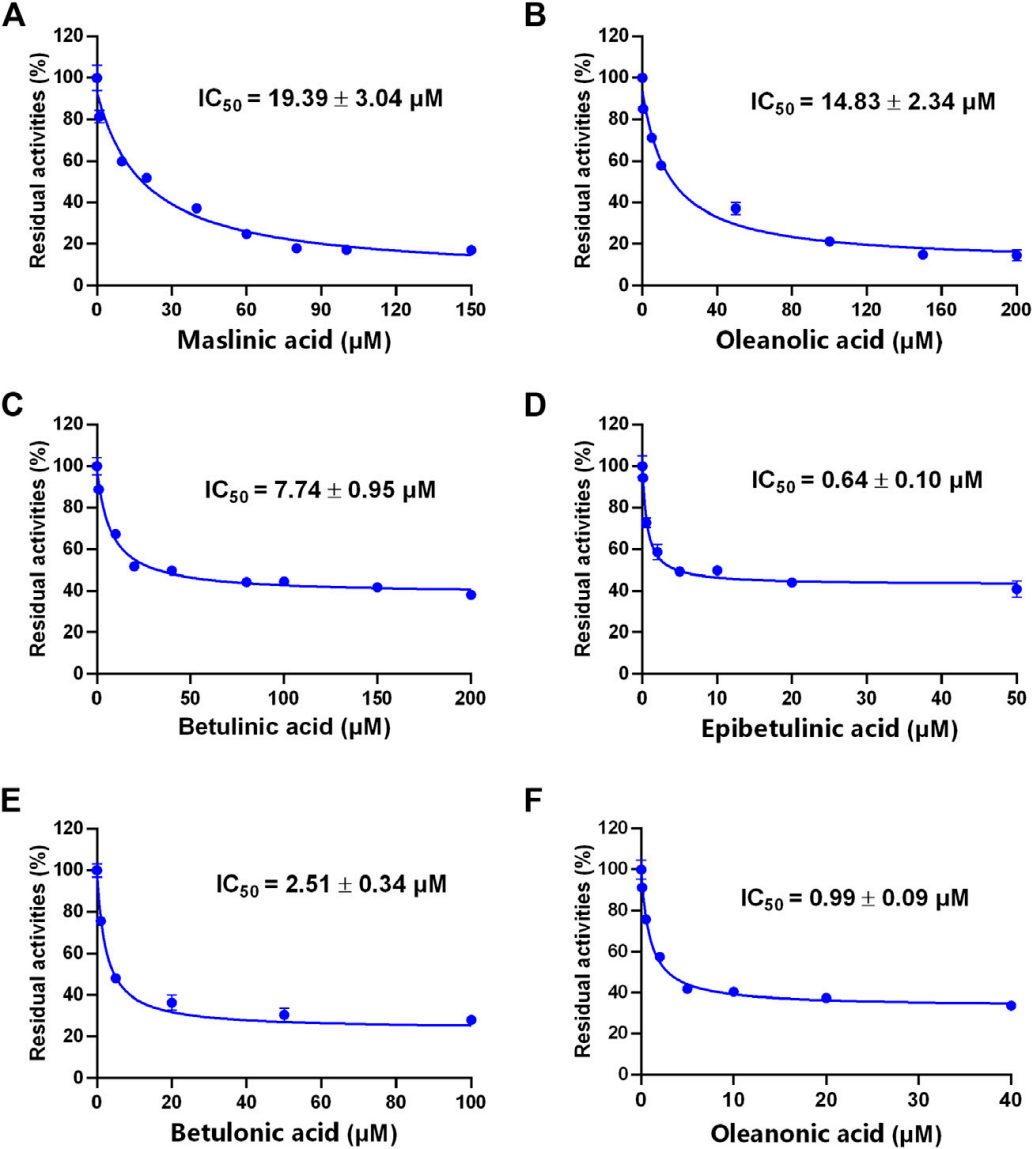
The inhibitory effects of the pentacyclic triterpenoid acids (maslinic acid **(A)**, oleanolic acid **(B)**, betulinic acid **(C)**, epibetulinic acid **(D)**, betulonic acid **(E)** and oleanonic acid **(F)**) against midazolam 1′-hydroxylation in HLMs. Data are expressed as mean ± SD.

**FIGURE 6:**
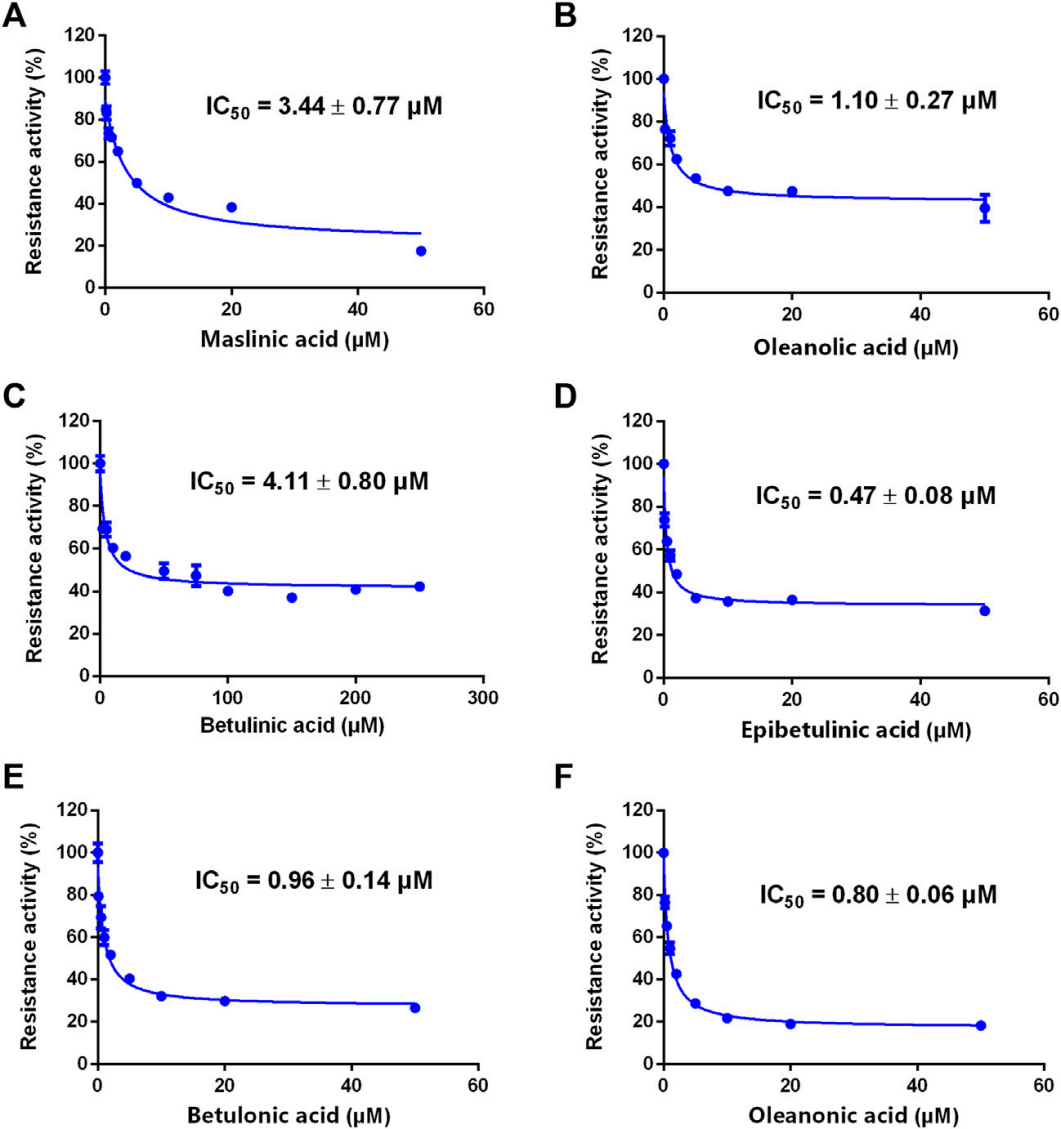
The inhibitory effects of the pentacyclic triterpenoid acids (maslinic acid **(A)**, oleanolic acid **(B)**, betulinic acid **(C)**, epibetulinic acid **(D)**, betulonic acid **(E)** and oleanonic acid **(F)**) against NEN-hydroxylation in HLMs. Data are expressed as mean ± SD.

A previous study revealed that the PTAs in Styrax displayed potent inhibition on CYP3A-catalyzed testosterone 6β-hydroxylation (Zhang et al., 2020). In this study, the inhibitory effects of these PTAs on CYP3A were assessed in HLMs by using a CYP3A-substrate drug (midazolam) and a newly reported CYP3A4 fluorescent substrate (NEN). As shown in Figures 5, 6 and Table 3, as well as Supplementary Figure S6, S7, the PTAs inhibited CYP3A-catalyzed midazolam 1′-hydroxylation in a dose-dependent manner, showing the IC_50_ values of 19.39, 32.20, 14.83, 7.74, 0.64, 2.51, and 0.99 μM, for maslinic acid, corosolic acid, oleanolic acid, betulinic acid, epibetulinic acid, betulonic acid and oleanonic acid, respectively. Meanwhile, all tested PTAs dose-dependently inhibited CYP3A4-catalyzed NEN 4-hydroxylation, with the IC_50_ values of 3.44, 41.93, 1.10, 4.11, 0.47, 0.96, and 0.80 μM, for maslinic acid, corosolic acid, oleanolic acid, betulinic acid, epibetulinic acid, betulonic acid and oleanonic acid, respectively. These results suggest that the PTAs in Styrax are key substances responsible for CYP3A inhibition, while epibetulinic acid, betulonic acid and oleanonic acid display potent CYP3A inhibitory effects.

**TABLE 3:** Inhibitory effects of pentacyclic triterpenoid acids on CYP3A in HLMs. Data are the mean ± SD.

Probe reaction	Compounds	Target enzyme	IC_50_ (μM)
Midazolam 1′-hydroxylation	maslinic acid	HLMs	19.39 ± 3.04
	corosolic acid	HLMs	32.20 ± 5.63
	oleanolic acid	HLMs	14.83 ± 2.34
	betulinic acid	HLMs	7.74 ± 0.95
	epibetulinic acid	HLMs	0.64 ± 0.10
	betulonic acid	HLMs	2.51 ± 0.34
	oleanonic acid	HLMs	0.99 ± 0.09
NEN 4-hydroxylation	maslinic acid	HLMs	3.44 ± 0.77
	corosolic acid	HLMs	41.93 ± 11.69
	oleanolic acid	HLMs	1.10 ± 0.27
	betulinic acid	HLMs	4.11 ± 0.80
	epibetulinic acid	HLMs	0.47 ± 0.08
	betulonic acid	HLMs	0.96 ± 0.14
	oleanonic acid	HLMs	0.80 ± 0.06

### Tissue distribution of seven PTAs from Styrax in rats

**FIGURE 7:**
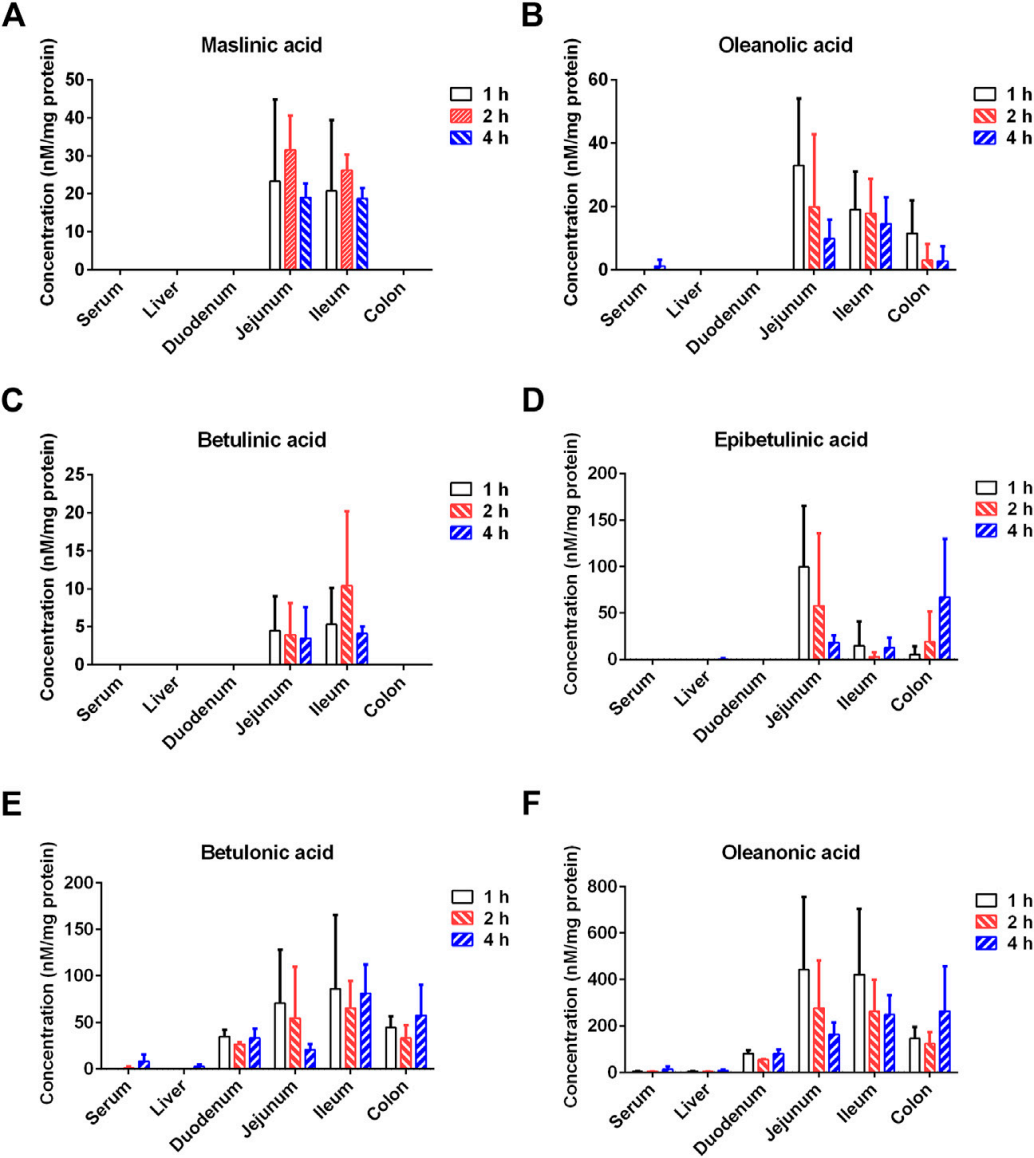
Tissue distribution of the major constituents of Styrax (maslinic acid **(A)**, oleanolic acid **(B)**, betulinic acid **(C)**, epibetulinic acid **(D)**, betulonic acid **(E)**, and oleanonic acid **(F)**) after a single oral dose of Styrax (100 mg/kg) to rats.

Next, the tissue distribution (in plasma, liver, duodenum, jejunum, ileum and colon) of seven PTAs from Styrax was determined in rats after single oral dose of Styrax (100 mg/kg). As shown in Figure 7, Supplementary Figure S8, and Supplementary Tables S9–S12, maslinic acid, corosolic acid and betulinic acid could not be detected (lower the detection limit) in rat plasma and rat liver, while very low exposure levels of epibetulinic acid, betulonic acid, oleanonic acid, oleanolic acid were detected in rat plasma and rat liver. By contrast, these PTAs could be easily detected from the intestinal tract (especially in jejunum, ileum and colon) in rats. Notably, the local exposure of oleanonic acid to rat jejunum could reach 745.69 nM/mg tissue protein (1 h), which was near to the IC_50_ value of oleanonic acid against CYP3A4. These results suggested that the major PTAs in Styrax could be distributed in intestinal tract at relatively high exposure levels but their plasma and liver exposure in rats was extremely low. These findings well explain why Styrax (i.g.) significantly elevates the plasma exposure of CYP3A-substrate drugs only when these agents are orally administrated, and why Styrax cannot modulate the pharmacokinetic behavior of CYP3A-substrate drugs when these agents administered intravenously.

## Discussion

Over the past few decades, the concomitant use of Chinese medicines and Western medicines has widely been used to cure a range of human diseases, such as digestive diseases, cardiovascular diseases, infectious diseases and various types of cancer (Klotz, 2009; Cong et al., 2015; Mair et al., 2020). In most cases, the concomitant use of Chinese medicines and Western medicines can bring better therapeutic effects *via* acting on various therapeutic targets or prolonging the half-lives of active compounds in either Chinese medicines or Western medicines (Wu et al., 2011; Shao et al., 2021; Zhang T. et al., 2022). However, in some cases, the concomitant use of Chinese medicines and Western medicines (especially for those agents with very narrow therapeutic windows) has a high risk of triggering potential HDIs (Meijerman et al., 2006; Han et al., 2020). It is well-known that most herbal medicines are administered orally, while Western medicines are administered in various ways including oral administration and injection. Oral drugs are generally absorbed through the mesentery, through the portal vein to the liver, and then enter into the blood circulation system. Thus, it should be clarified whether herbal medicines can modulate the pharmacokinetic behavior of Western medicine(s) by inhibiting/inactivating intestinal or hepatic drug-metabolizing enzymes *in vivo*.

As one of the most abundant drug-metabolizing enzymes (DMEs) in human liver and intestine, CYP3A4 plays a crucial role in the metabolic clearance and detoxification of a wide range of Western medicines *in vivo*, thus acts as a key mediator in HDIs and DDIs (Galetin et al., 2007; Zhou, 2008; Tian and Hu, 2014). In this study, following screening more than 100 kinds of clinical commonly used Chinese medicines, Styrax is found with the most potent CYP3A4 inhibitory effect (almost completely inhibit CYP3A4 at 100 μg/ml). Considering that Styrax and Styrax-related herbal products are frequently used in combination with a panel of marketed cardiovascular drugs (such as warfarin, digoxin and simvastatin) to treat cardiovascular diseases (Zhu, 2009; Tao et al., 2016; Yang et al., 2021). It is well-known that Styrax/Styrax-related herbal products are often taken orally for the treatment of human diseases, while a number of studies have reported that the local distribution of herbal constituents in the intestine is much higher than that in the liver (Lai et al., 2009; Wang et al., 2018). In these cases, it is necessary to clarify whether Styrax can trigger HDIs *in vivo* and to reveal the key organ(s) and molecular mechanisms involved in the pharmacokinetic HDIs triggered by Styrax.

The small intestine is the key organ for absorption, metabolism, and excretion of orally administered drugs in mammals (Yamashita et al., 2021). Many oral drugs can be metabolized by mammalian CYPs, while intestinal CYP3A4 makes a significant contribution to the metabolism of drugs (Galetin et al., 2007). In this study, *in vitro* assays demonstrated that Styrax and its major constituents (seven PTAs) potently inhibited CYP3A-catalyzed oxidative metabolism of various substrates. *In vivo* assays showed that Styrax only affected the pharmacokinetic behavior of oral victim CYP3A-substrate drugs (midazolam and felodipine), which prompted us to carefully evaluate the tissue distribution of Styrax/its active constituents (seven PTAs) after oral administration of this herbal extract in rats. These results clearly showed that seven PTAs from Styrax could be exposed to the intestinal tract (especially in jejunum, ileum and colon) at relatively high levels in rats after single oral dose of Styrax (100 mg/kg), but these PTAs were hardly exposed to the liver. The extremely low exposure of these PTAs from Styrax to mammal liver could be attributed to the poor oral bioavailability and the first-pass metabolism of PTAs (Zhang et al., 2019; Yin et al., 2022)*.* The high local exposure of PTAs in the intestinal tract indicated that intestinal CYP3A could be significantly inhibited by these natural compounds in Styrax, while the extremely low exposure of PTAs to mammal liver well-explained why Styrax hardly modulated the *in vivo* pharmacokinetic behaviors of the CYP3A-substrate drugs administrated by intravenous injection.

It should be noted that some CYP3A4 potent inhibitors (such as ritonavir) have been approved by FDA in combination with CYP3A4-substrate drugs to slow down the metabolic clearance of some important CYP3A4-substrate drugs *in vivo*, such as Aluvia/Kaletra (lopinavir and ritonavir) and paxlovid (nirmatrelvir and ritonavir) (Hurst and Faulds, 2000; Agarwal and Agarwal, 2021; Eng et al., 2022; Lamb, 2022). Thus, the potent CYP3A4 inhibitors with improved safety profiles could be developed as oral agents for improving the systemic exposure of CYP3A4-substrate drugs *in vivo*. Considering that hepatic CYP3A plays an important role in endogenous metabolism (such as bile acids and hormones) and the metabolic clearance of toxins, potent inhibition of hepatic CYP3A may lead to serious consequences (such as drug-induced liver injury and imbalance of hormone metabolism) (Li et al., 1995; Burk and Wojnowski, 2004; Banankhah et al., 2016; Qin et al., 2021). In this study, our findings show that the PTAs in Styrax are difficult to enter into the circulation system after oral administration of Styrax, Styrax or its main constituents (PTAs) can be intentionally used in combination with some CYP3A-substrate drugs with low oral bioavailability, aiming to improve its oral bioavailability without affecting the function of hepatic CYP3A. In future, the naturally occurring PTAs in Styrax could be used as promising lead compounds to develop more efficacious CYP3A inhibitors with strong anti-CYP3A effects, improved cell membrane permeability and high safety profiles.

## Conclusion

In summary, we demonstrate a case study to efficiently find the herbal medicine(s) with potent hCYP3A4 inhibitory effect *in vitro* and to accurately assess the potential HDIs risk *in vivo.* With the help of fluorescence-based high-throughput hCYP3A4 inhibition assay, the Chinese herb Styrax was found with the most potent hCYP3A4 inhibition in HLMs. *In vivo* pharmacokinetic assays demonstrated that Styrax (i.g., 100 mg/kg) only affected the pharmacokinetic behavior of orally administrated CYP3A-substrate drugs (midazolam and felodipine). Further investigations demonstrated that seven PTAs in Styrax were key substances responsible for CYP3A inhibition, while these PTAs could be exposed to intestinal tract at relatively high exposure levels but the exposure levels of these PTAs in rat plasma and liver were extremely low. Collectively, our findings showed that the Chinese herb Styrax (i.g.) could significantly elevate the plasma exposure of orally administrated CYP3A-substrate drugs but hardly modulated the pharmacokinetic profiles of intravenously administrated CYP3A-substrate drugs, which could be attributed to the high exposure levels of the PTAs in Styrax in intestinal tract and the extremely low exposure levels in rat liver. All these findings clearly suggest that the tissue exposure of some key constituents in herbal medicines strongly affect their *in vivo* inhibitory effects on CYP3A4, which are very helpful for the clinical pharmacologists to better understand the pharmacokinetic HDIs triggered by herbal products that contains naturally occurring CYP3A4 inhibitors.

## Data Availability

The raw data supporting the conclusions of this article will be made available by the authors, without undue reservation.

## Abbreviations

AUC, area under the plasma concentration; CMC-Na, sodium carboxymethyl cellulose; CYPs/P450s, cytochrome P450 enzymes; DDIs, drug-drug interactions; DMEs, drug-metabolizing enzymes; ESI, electrospray ionization; G-6-P, D-Glucose-6-phosphate; G-6-PDH, glucose-6-phosphate dehydrogenase; HDIs, herb-drug interactions; hCYP3A4, human cytochrome P450 3A4; HIMs, human intestine microsomes; HLMs, human liver microsomes; IC50, half maximal inhibition concentration; IS, internal standard; i.v., intravenous; i.g., intragastrical; MRM, multiple reaction monitoring; NEN, N-ethyl-1,8-naphthalimide; NEHN, N-ethyl-4-hydroxyl-1,8-naphthalimide; PBS, potassium phosphate buffer; PTAs, pentacyclic triterpenoid acids; RIMs, rat intestine microsomes; RLMs, rat liver microsomes; SHUTCM, Shanghai University of Traditional Chinese Medicine; t_1/2_, half-life; TICs, total ion chromatograms.
