# Supplementary material for: The Chinese herb Styrax triggers pharmacokinetic herb-drug interactions via inhibiting intestinal CYP3A
Source: Front Pharmacol. 2022 Aug 29;13:974578. doi: 10.3389/fphar.2022.974578 (PMC9469097; doi:10.3389/fphar.2022.974578)
Supplement: Supplementary file 1 [file DataSheet1.docx]

**Supplementary Materials**

*for*

**The Chinese herb Styrax triggers pharmacokinetic herb-drug interactions *via* inhibiting intestinal CYP3A**

Feng Zhang^a,b^, Tiantian Zhang^c^, Jiahao Gong^b^, Qinqin Fang^d^, Shenglan Qi^a,d^, Mengting Li^a^, Yan Han^a,*^, Wei Liu^d,*^, Guangbo Ge^b,*^

*^a^ Department of Neurology, Yueyang Hospital of Integrated Traditional Chinese and Western Medicine , Shanghai University of Traditional Chinese Medicine, Shanghai, China.*

*^b^ Shanghai Frontiers Science Center of TCM Chemical Biology; Institute of Interdisciplinary Integrative Medicine Research, Shanghai University of Traditional Chinese Medicine, Shanghai, 201203, China.*

*^c^ School of Pharmacy, Zunyi Medical University, Zunyi, Guizhou, China*

*^d^ Key Laboratory of Liver and Kidney Diseases (Ministry of Education), Institute of Liver Diseases, Shanghai Key Laboratory of Traditional Chinese Clinical Medicine, Shuguang Hospital Affiliated to Shanghai University of Traditional Chinese Medicine, Shanghai, China.*

^*^Corresponding authors:

Prof. Guang-Bo Ge, E-mail: [geguangbo@dicp.ac.cn](mailto:geguangbo@dicp.ac.cn)

Prof. Wei Liu, E-mail: lwhzayl@shutcm.edu.cn

Prof. Yan Han, E-mail: hanyan@shutcm.edu.cn

**This file contains twelve Tables and eight supplementary Figures.**

**Determination of 1'-hydroxymidazolam by LC-MS/MS**

The 1'-hydroxymidazolam were quantified using a Shimadzu UFLC system (Kyoto, Japan) combined with an AB Sciex QTrap 4500 tandem mass spectrometer (Foster City, CA, USA). The LC system consisted of a CBM-20A communication bus module, two LC-20AD pumps, a SIL-20AC autosampler and a CTO-20AC column oven. Chromatographic separation was performed on a C18 column (2.1 × 50 mm, 2.2 μm, Shimadzu) coupled with an ODS guard column (5 × 2.0 mm, 2.2 μm, Shimadzu), which was maintained at 40 °C with a flow rate of 0.4 mL/min. The injection volume was 2 μL and the total run time was 3.0 min. Water containing 0.1% (v/v) formic acid (solvent A) and acetonitrile (solvent B) were used as the mobile phases with following elution program: 0-0.50 min, 2% B; 0.50-0.60 min, 2-85% B; 0.60-1.60 min, 85% B; 1.60-1.70 min, 85-2% B; 1.70-3.00 min, 2% B. Electrospray ionization-tandem mass spectrometry (ESI-MS/MS) was used to quantify all analytes in both positive and negative ion modes, and the parameters are listed in **Table S2**.

**Analytical conditions of UHPLC-Q-Exactive Orbitrap HRMS**

An UHPLC-Q-Exactive Orbitrap system (Thermo Fisher Scientific Inc., Grand Island, NY, USA) was used to carry out the Chromatographic separation. The UHPLC system is composed of a Thermo Scientific Dionex Ultimate 3000 Series RS pump coupled with a Thermo Scientific Dionex Ultimate 3000 Series TCC-3000RS column compartments and WPS-3000 autosampler controlled by Chromeleon 7.2 Software. The cooling autosampler was set at 10 °C and protected from light, and the column heater was set at 40 °C. Method 1 (detected midazolam): A Waters ACQUITY UPLC BEH C_18_ column (2.1 × 50 mm, 1.7 μm) was used for Chromatographic separation, with a flow rate of 0.4 mL/min. The mobile phase was composed of A (acetonitrile) and B (0.1% formic acid), with the gradient as follows: 0-0.5 min (28% A), 0.5-2.0 min (28%-90% A), 2.0-3.0 min (90% A), 3.0-4.0 min (28% A). Method 2 (detected felodipine): A Waters ACQUITY UPLC BEH C_18_ column (2.1 × 50 mm, 1.7 μm) was used for Chromatographic separation, with a flow rate of 0.4 mL/min. The mobile phase was composed of A (acetonitrile) and B (0.1% formic acid), with the gradient as follows: 0-0.5 min (28% A), 0.5-1.2 min (28%-90% A), 1.2-3.0 min (90% A), 3.0-4.0 min (28% A). Method 3 (detected PTAs of Styrax): A Waters ACQUITY UPLC BEH C_18_ column (2.1 × 100 mm, 1.7 μm) was used to perform the Chromatographic separation, with a flow rate of 0.3 mL/min. The mobile phase was composed of A (acetonitrile) and B (0.1% formic acid), with the gradient as follows: 0-1.2 min (55% A), 1.2-7.2 min (55%-85% A), 7.2-7.3 min (85% A -95% A), 7.3-8.7 min (95% A), 8.7-10.0 min (55% A). Method 4 (identified chemical constituents of Styrax): A Waters ACQUITY UPLC BEH C_18_ column (2.1 × 100 mm, 1.7 μm) was used for Chromatographic separation, with a flow rate of 0.3 mL/min. The mobile phase was composed of A (methanol) and B (0.1% formic acid), with the gradient as follows: 0-1.0 min (4% A), 1.0-2.0 min (4%-20% A), 2.0-7.0 min (20% A -68% A), 7.0-21.0 min (68% A), 21.0-39.0 min (68% A-95% A), 39.0-43.5 min (95% A), 43.5-43.6 min (95% A-4% A), 43.6-45.0 min (4% A). The injection volume was 5 μL.

The mass spectrometer Q-Exactive Orbitrap system was connected to the UHPLC system *via* heated electrospray ionization (ESI) and controlled by Xcalibur 4.1 software that was used for data collection and analysis. The commonly parameters of mass spectrometry of four methods were: capillary temperature: 325°C; sheath gas (N_2_) flow rate: 45 arbitrary units; auxiliary gas (N_2_) flow rate: 8 arbitrary units; sweep gas flow rate: 0 arbitrary units; automatic gain control (AGC) 10^6^; s-lens RF level 50; auxilliary gas heater temperature, 300°C. The differential parameters of mass spectrometry of four methods were: Method 1 (detected midazolam, in positive ionization mode): spray voltage: 3.5 kV (positive); scan mode: Full MS/SIM mode: resolution: 70000 FWHM. Method 2 (detected felodipine, in negative ionization mode): spray voltage: 2.5 kV (negative); scan mode: Full MS/SIM mode: resolution: 70000 FWHM. Method 3 (detected PTAs of Styrax, in negative and positive ionization mode): spray voltage: 2.5 kV (negative), 3.5 kV (positive); scan mode: Full MS/SIM mode: resolution: 70000 FWHM. Method 4 (identified chemical constituents of Styrax, in negative and positive ionization mode): spray voltage: 2.5 kV (negative), 3.5 kV (positive); scan mode: Full MS/dd-MS^2^ mode, which includes 1 first-level full scan (resolution 70000 FWHM) and 1 data-dependent secondary scan (resolution 17500 FWHM) 2 events, the scanning range (*m/z*) is 80-1200, and the collision energy (CE) gradient is 20, 50, 100V. Mass spectrometry parameters were described in **Supplementary Figure S2-4** & **Supplementary Table S3-S5**.

**Table S1.** **The incubation and analytical conditions of probe substrate towards CYP3A4.**

| **Target enzyme** | **Substrate** | **Probe reactions** | **Substrate concentration (μM)** | **Enzyme concentration (μg/mL)** | **Detection conditions** |
| --- | --- | --- | --- | --- | --- |
| CYP3A4 | NEN | NEN 4-hydroxylation | 10 μM | 50 | Fluorescence  λ_ex/em_=450/558 nm (M) |

***M means the hydroxylated metabolite.**

**Table S2.** **Incubation and analytical conditions for quantification of 1'-hydroxymidazolam.**

| **Probe reactions/IS** | **Substrate concentration (μM)** | **Enzyme concentration (mg/mL)** | **Incubation time (min)** | **LC-MS/MS analysis for metabolite** | **ESI** | **DP (V)** | **CE (V)** |
| --- | --- | --- | --- | --- | --- | --- | --- |
| Midazolam 1’-hydroxylation | 5 | 0.1 | 30 | 341.9 > 324.1 | + | 100 | 30 |
| Lansoprazole (IS) |  |  |  | 370.1 > 252.0 | + | 100 | 17 |

**Table S3. Mass spectrometry parameters of midazolam and osalmide (internal standard, IS) in pharmacokinetic studies.**

| NO. | Target analytes | t_R_  /min | Ion mode | Formula | Calculated mass | Range of extracted mass |
| --- | --- | --- | --- | --- | --- | --- |
| 1 | 1’-OH-midazolam | 1.19 | [M+H]^+^ | C_18_H_13_ClFN_3_O | 342.0804 | 342.0770~342.0838 |
| 2 | midazolam | 1.30 | [M+H]^+^ | C_18_H_13_ClFN_3_ | 326.0855 | 326.0822~326.0888 |
| 3 | Osalmide (IS) | 1.70 | [M+H]^+^ | C_13_H_11_NO_3_ | 230.0812 | 230.0797~230.0825 |

**Table S4. Mass spectrometry parameters of felodipine and osalmide (internal standard, IS) in pharmacokinetic studies.**

| NO. | Target analytes | t_R_/min | Ion mode | Formula | Calculated mass | Range of extracted mass |
| --- | --- | --- | --- | --- | --- | --- |
| 1 | osalmide (IS) | 1.69 | [M-H]^-^ | C_13_H_11_NO_3_ | 228.0655 | 228.0641~228.0668 |
| 2 | felodipine | 2.35 | [M-H]^-^ | C_18_H_19_NO_4_Cl_2_ | 382.0607 | 382.0569~382.0646 |

**Table S5. Mass spectrometry parameters and contents of pentacyclic triterpenoid acids in Styrax.**

| NO. | Target analytes | t_R_/min | Ion mode | Formula | Calculated mass | Range of extracted mass | Contents (mg/g) |
| --- | --- | --- | --- | --- | --- | --- | --- |
| 1 | maslinic acid | 3.06 | [M-H]^-^ | C_30_H_48_O_4_ | 471.3469 | 471.3422~471.3516 | 14.80 |
| 2 | corosolic acid | 3.22 | [M-H]^-^ | C_30_H_48_O_4_ | 471.3469 | 471.3422~471.3516 | 1.92 |
| 3 | epibetulinic acid | 5.41 | [M-H]^-^ | C_30_H_48_O_3_ | 455.3520 | 455.3474~455.3565 | 1.42 |
| 4 | betulinic acid | 5.81 | [M-H]^-^ | C_30_H_48_O_3_ | 455.3520 | 455.3474~455.3565 | 3.54 |
| 5 | oleanolic acid | 6.18 | [M-H]^-^ | C_30_H_48_O_3_ | 455.3520 | 455.3474~455.3565 | 10.86 |
| 6 | betulonic acid | 7.04 | [M+H]^+^ | C_30_H_46_O_3_ | 455.3520 | 455.3474~455.3565 | 9.83 |
| 7 | oleanonic acid | 7.30 | [M+H]^+^ | C_30_H_46_O_3_ | 455.3520 | 455.3474~455.3565 | 34.19 |

**Table S6.** Inhibition effects of 119 botanical drug(s)/Chinese medicine granules/Chinese medicines (100 μg/mL, final concentration) against CYP3A4-catalyzed NEN-hydroxylation in HLMs.

| No. | Chinese medicines | Residual activity (%) |
| --- | --- | --- |
| - | Control | 100.00 ± 0.16 |
| 1 | Styrax [Liquidambar orientalis Mill.; Altingiaceae] | 9.25 ± 0.03 |
| 2 | *Radix Actinidiae Argutae* [Actinidia arguta (Siebold & Zucc.) Planch. ex Miq.; Actinidiaceae] | 24.98 ± 6.57 |
| 3 | *Herba Duchesneae Indicae* [Potentilla indica (Andrews) Th.Wolf; Rosaceae] | 31.53 ± 0.41 |
| 4 | *Lycopi Herba* [Lycopus lucidus Turcz. ex Benth.; Lamiaceae] | 32.75 ± 10.68 |
| 5 | *Herba Leonuri* [Leonurus japonicus Houtt.; Lamiaceae] | 32.93 ± 3.39 |
| 6 | *Pericarpium Zanthoxyli* [Zanthoxylum bungeanum Maxim.; Rutaceae] | 34.05 ± 1.07 |
| 7 | *Blackend Swallowwort Root* [Cynanchum atratum Bunge; Apocynaceae] | 34.20 ± 1.03 |
| 8 | *Radix glycyrrhizae preparata* [Glycyrrhiza uralensis Fisch. ex DC.; Fabaceae] | 34.98 ± 2.24 |
| 9 | *Radix oryzae glutinosae* [Oryza sativa L.; Poaceae] | 36.00 ± 2.43 |
| 10 | *Pubescent Holly Root* [Ilex pubescens Hook. & Arn.; Aquifoliaceae] | 39.63 ± 3.25 |
| 11 | *Fructus Tsaoko* [Lanxangia tsao-ko (Crevost & Lemarié) M.F.Newman & Skornick.; Zingiberaceae] | 41.21 ± 17.27 |
| 12 | *Angelicae Dahuricae Radix* [Angelica dahurica (Hoffm.) Benth. & Hook.f. ex Franch. & Sav.; Apiaceae] | 42.16 ± 1.99 |
| 13 | *Epimedii Folium* [Epimedium brevicornu Maxim.; Berberidaceae] | 44.14 ± 3.47 |
| 14 | *Andrographis Herba* [Andrographis paniculata (Burm.f.) Nees; Acanthaceae] | 46.61 ± 8.09 |
| 15 | *Semen Trigonellae* [Trigonella foenum-graecum L.; Fabaceae] | 46.70 ± 3.82 |
| 16 | *Liquidambaris Fructus* [Liquidambar formosana Hance; Altingiaceae] | 46.76 ± 1.34 |
| 17 | *Rhizoma Curcumae* [Curcuma aeruginosa Roxb.; Zingiberaceae] | 47.48 ± 3.23 |
| 18 | *Cnidii Fructus* [Cnidium monnieri (L.) Cusson; Apiaceae] | 48.39 ± 0.93 |
| 19 | *Inulae Flos* [Inula japonica Thunb.; Asteraceae] | 49.74 ± 8.33 |
| 20 | *Linderae Radix* [Lindera aggregata (Sims) Kosterm.; Lauraceae] | 50.16 ± 8.86 |
| 21 | *Aesculi Semen* [Aesculus chinensis Bunge; Sapindaceae] | 51.82 ± 1.19 |
| 22 | *Arnebiae Radix* [Lithospermum erythrorhizon Siebold & Zucc.; Boraginaceae] | 52.00 ± 1.76 |
| 23 | *Herba Polygoni Avicularis* [Polygonum aviculare L.; Polygonaceae] | 54.20 ± 4.20 |
| 24 | *Buddlejae Flos* [Buddleja officinalis Maxim.; Scrophulariaceae] | 57.21 ± 4.83 |
| 25 | *oriental waterplantain rhizome* [Alisma plantago-aquatica L.; Alismataceae] | 58.64 ± 8.10 |
| 26 | *Trachlospermi Caulis Et Folium* [Trachelospermum jasminoides (Lindl.) Lem.; Apocynaceae] | 60.38 ± 4.84 |
| 27 | *Pogostemonis Herba* [Pogostemon cablin (Blanco) Benth.; Lamiaceae] | 60.10 ± 3.15 |
| 28 | *Sophorae Flos* [Styphnolobium japonicum (L.) Schott; Fabaceae] | 62.47 ± 0.83 |
| 29 | *Alpiniae Oxyphyllae Fructus* [Alpinia oxyphylla Miq.; Zingiberaceae] | 62.74 ± 8.73 |
| 30 | *Euodiae Fructus* [Tetradium ruticarpum (A.Juss.) T.G.Hartley; Rutaceae] | 64.85 ± 4.09 |
| 31 | *Citrus reticulata Blanco* [Citrus aurantium L.; Rutaceae] | 64.91 ± 5.23 |
| 32 | *Houttuynia cordata Thunb* [Houttuynia cordata Thunb.; Saururaceae] | 66.55 ± 10.42 |
| 33 | *Eriobotryae Folium* [Eriobotrya japonica Thunb.; Rosaceae] | 69.89 ± 3.67 |
| 34 | *Fructus Piperis Longi* [Piper longum L.; Piperaceae] | 70.05 ± 8.50 |
| 35 | *Wenyujin Rhizoma Concisum* [Curcuma aromatica Salisb.; Zingiberaceae] | 70.97 ± 0.95 |
| 36 | *Arecae Pericarpium* [Areca catechu L.; Arecaceae] | 71.42 ± 1.07 |
| 37 | *Plantaginis Herba* [Plantago depressa Willd.; Plantaginaceae] | 72.88 ± 3.81 |
| 38 | *Corydalis Rhizoma* [Corydalis yanhusuo (Y.H.Chou & Chun C.Hsu) W.T.Wang ex Z.Y.Su & C.Y.Wu; Papaveraceae] | 73.49 ± 9.64 |
| 39 | *Leaf of Leatherleaf Mahonia* [Berberis bealei Fortune; Berberidaceae] | 73.92 ± 8.16 |
| 40 | *Dendrobii Caulis* [Dendrobium nobile Lindl.; Orchidaceae] | 73.94 ± 1.56 |
| 41 | *Angelicae Pubescentis Radix* [Heracleum hemsleyanum Diels; Apiaceae] | 75.01 ± 1.02 |
| 42 | *Rhizoma Zingiberis Preparata* [Zingiber officinale Roscoe; Zingiberaceae] | 75.30 ± 5.51 |
| 43 | *Nutgrass Galingale Rhizome* [Cyperus rotundus L.; Cyperaceae] | 76.09 ± 0.73 |
| 44 | *Albiziae Cortex* [Albizia julibrissin Durazz.; Fabaceae] | 77.34 ± 8.54 |
| 45 | *Schizonepetae Herba* [Nepeta cataria L.; Lamiaceae] | 77.44 ± 2.76 |
| 46 | *Pericarpium Trichosanthis* [Trichosanthes kirilowii Maxim.; Cucurbitaceae] | 78.37 ± 7.11 |
| 47 | *Sepiae Endoconcha* | 86.16 ± 15.19 |
| 48 | *Medulla Junci* [Juncus effusus L.; Juncaceae] | 78.73 ± 2.66 |
| 49 | *Faeces Trogopteri* | 78.75 ± 3.01 |
| 50 | *Dipsaci Radix* [Dipsacus asper Wall. ex DC.; Caprifoliaceae] | 78.86 ± 8.60 |
| 51 | *Pericarpium Citri Reticulatae Viride* [Citrus aurantium L.; Rutaceae] | 78.95 ± 6.22 |
| 52 | *Homalomenae Rhizoma* [Homalomena occulta (Lour.) Schott; Araceae] | 78.96 ± 8.69 |
| 53 | *Nelumbinis Semen* [Nelumbo nucifera Gaertn.; Nelumbonaceae] | 79.02 ± 10.96 |
| 54 | *Campsis Flos* [Campsis grandiflora (Thunb.) K.Schum.; Bignoniaceae] | 79.56 ± 1.65 |
| 55 | *Eucommiae Cortex* [Eucommia ulmoides Oliv.; Eucommiaceae] | 79.88 ± 8.97 |
| 56 | *Herba Artemisiae Scopariae* [Artemisia capillaris Thunb.; Asteraceae] | 80.65 ± 7.44 |
| 57 | *Lasiosphaera* | 80.66 ± 1.92 |
| 58 | *Fructus Malvae Verticillatae* [Malva verticillata var. Verticillata; Malvaceae] | 81.21 ± 6.41 |
| 59 | *Rehmanniae Radix Praeparata* [Rehmannia glutinosa (Gaertn.) DC.; Orobanchaceae] | 81.90 ± 1.33 |
| 60 | *Dark Plum Fruit* [Prunus mume (Siebold) Siebold & Zucc.; Rosaceae] | 82.10 ± 7.58 |
| 61 | *Radix Rhapontici* [Leuzea uniflora (L.) Holub; Asteraceae] | 82.96 ± 0.71 |
| 62 | *Commelinae Herba* [Commelina communis L.; Commelinaceae] | 83.16 ± 9.18 |
| 63 | *Ramulus Mori* [Morus alba L.; Moraceae] | 84.90 ± 1.65 |
| 64 | *Chrysanthemi Flos* [Chrysanthemum × morifolium (Ramat.) Hemsl.; Asteraceae] | 84.96 ± 3.72 |
| 65 | *CommonClubmoss Herb* [Lycopodium japonicum Thunb.; Lycopodiaceae] | 86.27 ± 1.63 |
| 66 | *Pinelliae Rhizoma* [Pinellia ternata (Thunb.) Makino; Araceae] | 86.33 ± 6.12 |
| 67 | *Magnoliae Officmalis Flos* [Magnolia officinalis Rehder & E.H.Wilson; Magnoliaceae] | 87.15 ± 6.55 |
| 68 | *Arisaematis Rhizoma* [Arisaema heterophyllum Blume; Araceae] | 87.55 ± 3.03 |
| 69 | *Aconiti Radix* [Aconitum carmichaeli Debeaux; Ranunculaceae] | 88.65 ± 7.12 |
| 70 | *Cirsii Japonici Herba Carbonisata* [Cirsium japonicum DC.; Asteraceae] | 88.82 ± 1.28 |
| 71 | *Flower of Hyacinth Dolichos* [Lablab purpureus (L.) Sweet; Fabaceae] | 89.08 ± 11.28 |
| 72 | *Polygonati Rhizoma* [Polygonatum sibiricum Redouté; Asparagaceae] | 89.21 ± 5.73 |
| 73 | *Aconiti Lateralis Radix Praeparata* [Aconitum carmichaeli Debeaux; Ranunculaceae] | 89.30 ± 4.62 |
| 74 | *Ampelopsis Radix* [Ampelopsis japonica (Thunb.) Makino; Vitaceae] | 90.38 ± 6.29 |
| 75 | *Persicae Semen* [Prunus persica (L.) Batsch; Rosaceae] | 90.47 ± 7.93 |
| 76 | *Galli Gigerii Endothelium Corneum* | 90.74 ± 7.06 |
| 77 | *Citri Reticulatae Semen* [Citrus aurantium L.; Rutaceae] | 90.76 ± 7.80 |
| 78 | *Tangerine Pith* [Citrus aurantium L.; Rutaceae] | 91.35 ± 2.34 |
| 79 | *Spirodelae Herba* [Spirodela polyrrhiza (L.) Schleid.; Araceae] | 91.35 ± 6.63 |
| 80 | *Portulacae Herba* [Portulaca oleracea L.; Portulacaceae] | 91.63 ± 10.61 |
| 81 | *Haematitum* | 92.01 ± 3.97 |
| 82 | *Eupatorii Herba* [Eupatorium fortunei Turcz.; Asteraceae] | 92.15 ± 8.84 |
| 83 | *Peucedani Radix* [Peucedanum praeruptorum Dunn; Apiaceae] | 92.60 ± 3.17 |
| 84 | *Thallus Laminariae* | 93.14 ± 5.42 |
| 85 | *Arisaema Cum Bile* [Arisaema heterophyllum Blume; Araceae] | 93.16 ± 6.23 |
| 86 | *Irkutsk Anemone Rhizome* [Anemonoides altaica (Fisch. ex C.A.Mey.) Holub; Ranunculaceae] | 93.19 ± 6.01 |
| 87 | *Paridis Rhizoma* [Paris polyphylla Sm.; Melanthiaceae] | 93.36 ± 1.56 |
| 88 | *Schisandrae Chinensis Fructus* [Schisandra chinensis (Turcz.) Baill.; Schisandraceae] | 93.36 ± 3.40 |
| 89 | *Astmgali Radix* [Astragalus mongholicus Bunge; Fabaceae] | 93.71 ± 0.66 |
| 90 | *Ginkgo Semen* [Ginkgo biloba L.; Ginkgoaceae] | 94.79 ± 2.27 |
| 91 | *Poria* | 95.27 ± 1.26 |
| 92 | *Radix Trichosanthis* [Trichosanthes kirilowii Maxim.; Cucurbitaceae] | 96.05 ± 0.88 |
| 93 | *Angelicae Sinensis Radix* [Angelica sinensis (Oliv.) Diels; Apiaceae] | 96.56 ± 7.16 |
| 94 | *Chloriti Lapis* | 96.76 ± 7.70 |
| 95 | *Farfarae Flos* [Tussilago farfara L.; Asteraceae] | 96.81 ± 1.49 |
| 96 | *Citri Fructus* [Citrus medica L.; Rutaceae] | 97.28 ± 9.77 |
| 97 | *Pruni Semen* [Prunus japonica Thunb.; Rosaceae] | 97.48 ± 3.52 |
| 98 | *Foeniculi Fructus* [Foeniculum vulgare Mill.; Apiaceae] | 97.99 ± 7.96 |
| 99 | *Morindae Officinalis Radix* [Gynochthodes officinalis (F.C.How) Razafim. & B.Bremer; Rubiaceae] | 98.26 ± 8.72 |
| 100 | *Os Costaziae* | 98.28 ± 1.90 |
| 101 | *Akebiae Caulis* [Akebia quinata (Thunb. ex Houtt.) Decne.; Lardizabalaceae] | 98.64 ± 6.82 |
| 102 | *Saposhnikoviae Radix* [Saposhnikovia divaricata (Turcz. ex Ledeb.) Schischk.; Apiaceae] | 98.84 ± 6.19 |
| 103 | *Stachyuri Medulla* [Stachyurus himalaicus Hook.f. & Thomson ex Benth.; Stachyuraceae] | 98.98 ± 5.07 |
| 104 | *Hordei Fructus Germinatus* [Hordeum vulgare L.; Poaceae] | 99.14 ± 7.21 |
| 105 | *Phragmitis Rhizoma* [Phragmites australis subsp. Australis; Poaceae] | 99.33 ± 2.15 |
| 106 | *Rhizoma Dioscoreae* [Dioscorea polystachya Turcz.; Dioscoreaceae] | 99.56 ± 4.03 |
| 107 | *Imperatae Rhizoma* [Imperata cylindrica (L.) P.Beauv.; Poaceae] | 99.76 ± 3.92 |
| 108 | *Ginseng Radix Et Rhizoma* [Panax ginseng C.A.Mey.; Araliaceae] | 99.76 ± 8.36 |
| 109 | *Bulb of Thunberg Fritillary* [Fritillaria thunbergii Miq.; Liliaceae] | 99.83 ± 0.64 |
| 110 | *Concha Meretricis Seu Cyclinae* | 100.31 ± 2.43 |
| 111 | *Platycodonis Radix* [Platycodon grandiflorus (Jacq.) A.DC.; Campanulaceae] | 100.90 ± 0.10 |
| 112 | *Aconiti Kusnezoffii Radix* [Aconitum kusnezoffii Rchb.; Ranunculaceae] | 103.02 ± 8.56 |
| 113 | *Ophicalcitum* | 103.47 ± 6.59 |
| 114 | *Gypsum Fibrosum* | 106.20 ± 0.46 |
| 115 | *Indian Bread* with Pine | 106.34 ± 10.21 |
| 116 | *Euryales Semen* [Euryale ferox Salisb.; Nymphaeaceae] | 107.98 ± 5.44 |
| 117 | *Lilii Bulbus* [Lilium brownii var. Viridulum Baker; Liliaceae] | 109.69 ± 2.45 |
| 118 | *Bambusae Concretio Silicea* [Bambusa textilis McClure; Poaceae] | 110.19 ± 2.00 |
| 119 | *Typhonii Rhizoma* [Sauromatum giganteum (Engl.) Cusimano & Hett.; Araceae] | 113.03 ± 8.88 |

**Table S7. Inhibitory effects of Styrax on Midazolam 1’-hydroxylation in HLMs, HIMs, RLMs, RIMs. Data are the mean ± SD.**

| **Probe reaction** | **Enzyme Sources** | **IC_50_ (μg/mL)** |
| --- | --- | --- |
| Midazolam 1’-hydroxylation | HLMs | 3.72 ± 0.76 |
| Midazolam 1’-hydroxylation | HIMs | 2.84 ± 0.32 |
| Midazolam 1’-hydroxylation | RLMs | 2.90 ± 0.26 |
| Midazolam 1’-hydroxylation | RIMs | 1.67 ± 0.12 |

**Table S8. Ingredient identification of Styrax by UHPLC-Q-Exactive Orbitrap HRMS.**

| NO. | RT  /min | Ion mode | Measured/Da | Calculated/Da | Error  /ppm | Formula | Identification | MS/MS |
| --- | --- | --- | --- | --- | --- | --- | --- | --- |
| 1^*^ | 4.62 | [M-H]^-^ | 121.02855 | 121.02840 | 1.190 | C_7_H_6_O_2_ | Benzoic acid | 121.02863, 94.02881, 91.37063 |
| 2^*^ | 4.91 | [M+H]^+^ | 153.05528 | 153.05462 | 4.308 | C_8_H_8_O_3_ | Vanillin | 153.05536, 125.06046, 111.04493, 93.03448, 65.03967 |
| 3^*^ | 5.48 | [M+H]^+^ | 147.04445 | 147.04406 | 2.679 | C_9_H_8_O_2_ | Cinnamic acid | 147.04477, 119.04998, 91.05527 |
| 4 | 6.36 | [M+H]^+^ | 151.07600 | 151.07536 | 4.262 | C_9_H_10_O_2_ | 3-Phenylpropionic acid | 151.0624, 137.0303, 123.08131, 105.0709 |
| 5 | 12.84 | [M+H]^+^ | 133.06535 | 133.06479 | 4.197 | C_9_H_8_O | Cinnamaldehyde | 133.0654, 107.0500, 91.0552 |
| 6 | 14.47 | [M+Na]^+^ | 261.08948 | 261.08860 | 3.367 | C_16_H_14_O_2_ | Benzyl cinnamate | 261.0895, 117.07072, 88.02255 |
| 7 | 18.28 | [M+H]^+^ | 381.18622 | 381.18491 | 3.446 | C_27_H_24_O_2_ | Unknow | 381.1861, 155.0856, 129.0700, 117.0700 |
| 8 | 19.15 | [M+H]^+^ | 267.13885 | 267.13796 | 3.345 | C_18_H_18_O_2_ | 3-Phenylpropyl cinnamate | 149.0606, 131.0499, 119.0864, 103.0551, 91.0552 |
| 9^*^ | 28.37 | [M-H]^-^ | 471.3501 | 471.34689 | 6.818 | C_30_H_48_O_4_ | Maslinic acid | 471.3273, 425.3431, 407.3327, 355.2643, 205.1596 |
| 10^*^ | 29.03 | [M-H]^-^ | 471.34991 | 471.34689 | 6.415 | C_30_H_48_O_4_ | Corosolic acid | 471.3273, 425.3433, 407.3341, 355.2643 205.1596 |
| 11^*^ | 29.85 | [M-H]^-^ | 455.35507 | 455.35197 | 6.804 | C_30_H_48_O_3_ | Epibetulinic acid | 455.3542, 437.3510, 391.3515 |
| 12 | 30.05 | [M-H]^-^ | 471.35001 | 471.34689 | 6.627 | C_30_H_48_O_4_ | 3α, 25-Dihydroxy-olean-12-en-28-oic acid | 471.3273, 425.3429, 409.3481, 391.3347, 203.1804, 189.1647, 177.1646 |
| 13 | 30.56 | [M+H]^+^ | 471.34836 | 471.34689 | 3.126 | C_30_H_46_O_4_ | 3-oxo-12α-hydroxy-olean-28,13β-olide or isomer | 471.3273, 453.3388, 425.3404, 407.3324, 205.1597, 187.1490, 179.1438 |
| 14 | 31.63 | [M+H]^+^ | 471.3483 | 471.34689 | 2.999 | C_30_H_46_O_4_ | 3-oxo-12α-hydroxy-olean-28,13β-olide or isomer | 471.3273, 453.3415, 425.3431, 407.3326, 203.1804, 189.1648, 177.1646 |
| 15^*^ | 33.01 | [M-H]^-^ | 455.35501 | 455.35197 | 6.672 | C_30_H_48_O_3_ | Betulinic acid | 455.3542, 437.3510, 409.3634, 391.3531, 205.1596 |
| 16^*^ | 33.88 | [M-H]^-^ | 455.35501 | 455.35197 | 6.672 | C_30_H_48_O_3_ | Oleanolic acid | 455.3542, 437.3521, 409.3643, 391.3534, 205.1596 |
| 17 | 34.01 | [M+H]^+^ | 515.37482 | 515.37310 | 3.335 | C_32_H_50_O_5_ | 3α-acetoxy-25-hydroxy-olean-12-en-28-oic acid | 515.3749, 437.3440, 409.3478, 391.3385, 381.3541, 279.1966, 203.1804, 189.1646 |
| 18 | 34.42 | [M+H]^+^ | 303.23279 | 303.23186 | 3.078 | C_20_H_30_O_2_ | Abietic acid | 303.3058, 247.2440, 219.2120, 177.1642, 149.1333, 135.1176 |
| 19^*^ | 34.78 | [M+H]^+^ | 455.35318 | 455.35197 | 2.653 | C_30_H_46_O_3_ | Betulonic acid | 455.3521, 409.3484, 327.2316, 203.1803, 177.1646 |
| 20 | 34.92 | [M+H]^+^ | 471.34839 | 471.34689 | 3.190 | C_30_H_46_O_4_ | 3-oxo-12α-hydroxy-olean-28,13β-olide or isomer | 471.3273, 435.3279, 425.3400, 407.3326, 389.3211, 201.1647 |
| 21^*^ | 35.25 | [M+H]^+^ | 455.35318 | 455.35197 | 2.653 | C_30_H_46_O_3_ | Oleanonic acid | 455.3521, 437.3426, 409.3483, 391.3378, 203.1804 |

*-is expressed as confirmed by reference substance; RT-retention time

**Table S9. Results of linear regression, correlation coefficient, liner range and LLOQ of target pentacyclic triterpenoid acids.**

| **Target analytes** | **linear regression** | **R^2^** | **Linear range (nmol/L)** | **LLOQ**  **(nmol/L)** |
| --- | --- | --- | --- | --- |
| maslinic acid | Y = 112488X-19529 | 0.9999 | 8.19-320.00 | 8.19 |
| corosolic acid | Y = 112799X-58343 | 1.0000 | 8.19-320.00 | 8.19 |
| epibetulinic acid | Y = 194723X-255731 | 0.9997 | 3.28-128.00 | 3.28 |
| betulinic acid | Y = 239023X-91134 | 0.9999 | 3.28-128.00 | 3.28 |
| oleanolic acid | Y = 54277X-68953 | 0.9999 | 3.28-128.00 | 3.28 |
| betulonic acid | Y = 443601X-466519 | 0.9995 | 3.28-128.00 | 3.28 |
| oleanonic acid | Y = 399964X+164759 | 0.9993 | 3.28-128.00 | 3.28 |

**Table S10. Results of precision, accuracy, matrix effect and extraction recovery of target pentacyclic triterpenoid acids**

| **Target analytes** | **Nominal level (nmol/L)** | **Precision RSD%** | | **Accuracy %** | **Matrix effect %** | **Recovery/%** |
| --- | --- | --- | --- | --- | --- | --- |
|  |  | **Intra-day** | **Inter-day** |  |  |  |
| maslinic acid | 8.19 | 0.91 | 6.56 | 93.75 | 109.42 | 105.89 |
|  | 51.20 | 8.35 | 1.96 | 100.80 | 103.69 | 105.61 |
|  | 320.00 | 4.74 | 4.85 | 101.10 | 100.84 | 102.27 |
| corosolic acid | 8.19 | 0.91 | 6.56 | 97.69 | 108.99 | 105.22 |
|  | 51.20 | 8.35 | 1.96 | 101.20 | 103.66 | 104.81 |
|  | 320.00 | 4.74 | 4.85 | 100.93 | 100.84 | 102.26 |
| epibetulinic acid | 3.28 | 14.22 | 6.28 | 113.91 | 106.91 | 97.76 |
|  | 20.48 | 7.61 | 2.92 | 98.76 | 111.81 | 100.32 |
|  | 128.00 | 7.44 | 4.39 | 103.12 | 114.60 | 104.02 |
| betulinic acid | 3.28 | 11.31 | 5.76 | 104.78 | 103.64 | 102.66 |
|  | 20.48 | 13.59 | 4.79 | 99.37 | 106.94 | 101.65 |
|  | 128.00 | 3.86 | 9.65 | 101.99 | 111.25 | 105.99 |
| oleanolic acid | 3.28 | 8.48 | 6.59 | 113.93 | 108.56 | 107.84 |
|  | 20.48 | 9.27 | 3.99 | 103.87 | 112.41 | 97.33 |
|  | 128.00 | 4.81 | 3.31 | 103.35 | 114.29 | 105.83 |
| betulonic acid | 3.28 | 9.87 | 5.70 | 109.07 | 109.29 | 98.30 |
|  | 20.48 | 9.85 | 1.27 | 102.81 | 111.83 | 94.09 |
|  | 128.00 | 4.66 | 3.71 | 105.06 | 111.49 | 98.99 |
| oleanonic acid | 3.28 | 11.50 | 9.43 | 88.07 | 107.57 | 105.51 |
|  | 20.48 | 11.35 | 7.19 | 101.63 | 110.50 | 102.18 |
|  | 128.00 | 5.67 | 8.47 | 103.06 | 113.40 | 114.24 |

**Table S11. Results of stability tests for target pentacyclic triterpenoid acids.**

| **Target analytes** | **Nominal level (nmol/L)** | **Room temperature (24 h, %)** | **4 ℃ Refrigerator (3 day, %)** | **-20℃ freeze-thaw (3 times, %）** |
| --- | --- | --- | --- | --- |
| maslinic acid | 8.19 | 95.84 | 101.20 | 100.54 |
|  | 51.20 | 99.51 | 103.76 | 100.05 |
|  | 320.00 | 96.85 | 97.98 | 98.47 |
| corosolic acid | 8.19 | 99.78 | 105.13 | 104.46 |
|  | 51.20 | 99.91 | 104.14 | 100.45 |
|  | 320.00 | 96.69 | 97.82 | 98.31 |
| epibetulinic acid | 3.28 | 104.88 | 113.34 | 109.83 |
|  | 20.48 | 96.59 | 105.44 | 106.94 |
|  | 128.00 | 106.30 | 107.19 | 110.52 |
| betulinic acid | 3.28 | 98.14 | 100.20 | 104.81 |
|  | 20.48 | 102.89 | 102.78 | 99.04 |
|  | 128.00 | 103.89 | 102.12 | 110.33 |
| oleanolic acid | 3.28 | 103.28 | 105.69 | 103.79 |
|  | 20.48 | 99.64 | 102.19 | 105.51 |
|  | 128.00 | 99.00 | 104.96 | 105.16 |
| betulonic acid | 3.28 | 106.38 | 114.23 | 113.48 |
|  | 20.48 | 98.54 | 111.21 | 102.73 |
|  | 128.00 | 101.75 | 107.11 | 107.06 |
| oleanonic acid | 3.28 | 86.92 | 85.84 | 91.69 |
|  | 20.48 | 101.86 | 103.95 | 109.44 |
|  | 128.00 | 98.85 | 100.64 | 113.69 |

**Table S12. The structure of active compounds.**

| **Compound** | **Structure** | **MW** |
| --- | --- | --- |
| betulinic acid |  | 456.7 |
| oleanonic acid |  | 454.7 |
| epibetulinic acid |  | 456.7 |
| betulonic acid |  | 454.7 |
| maslinic acid |  | 472.7 |
| corosolic acid |  | 472.7 |
| oleanolic acid |  | 456.7 |





**Figure S1. Distribution of CYP3A4 in various tissues *in vivo.***


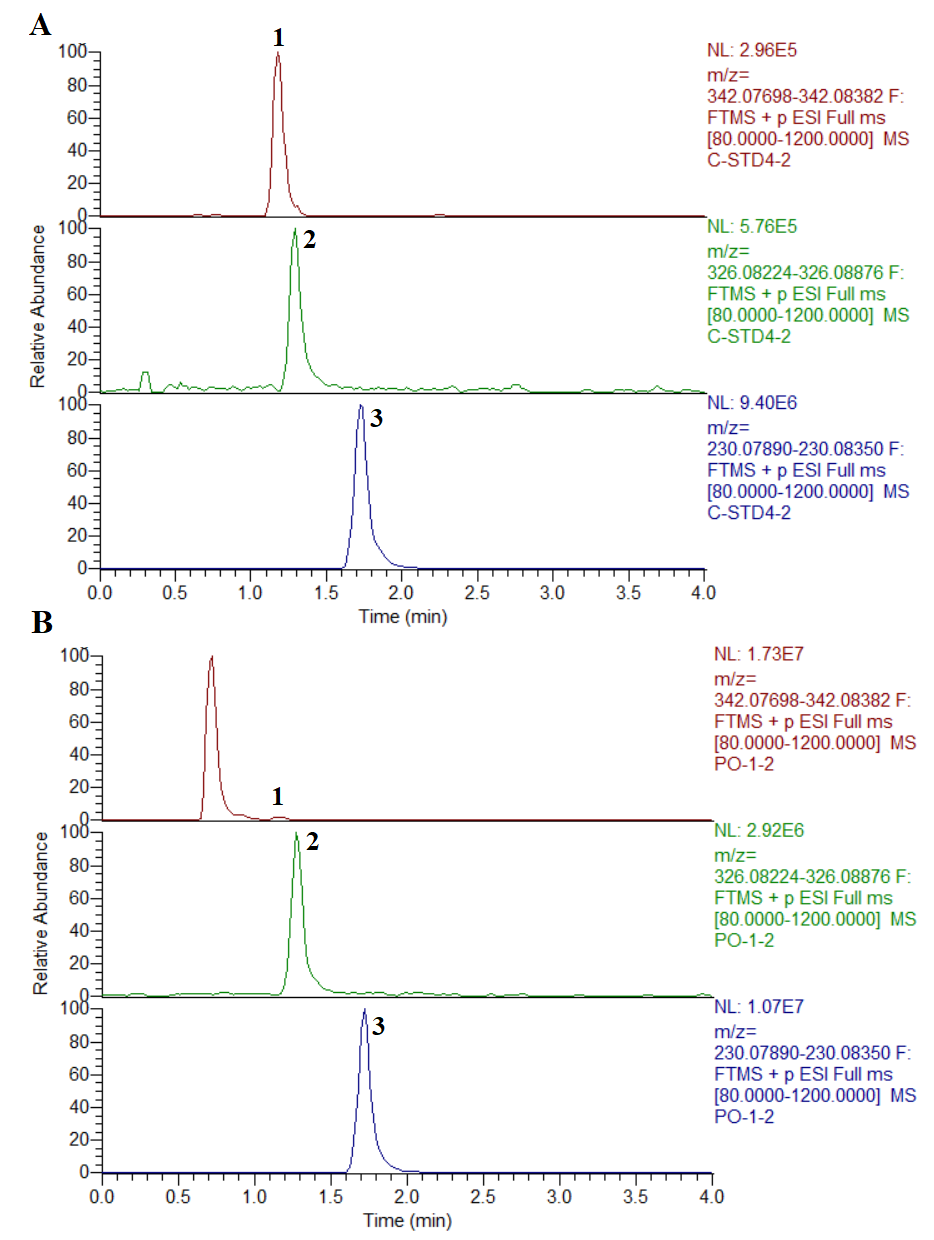


**Figure S2. UHPLC-Q-Orbitrap HRMS chromatograms of 1’-OH-midazolam, midazolam and osalmide (internal standard, IS). (**A) a blank plasma spiked with the analytes (with LLOQ) and IS (150.00 ng/mL). (B) a plasma sample (5 min) from a rat after oral administration of 20 mg/kg midazolam. 1, 1’-OH-midazolam; 2, midazolam; 3, osalmide.


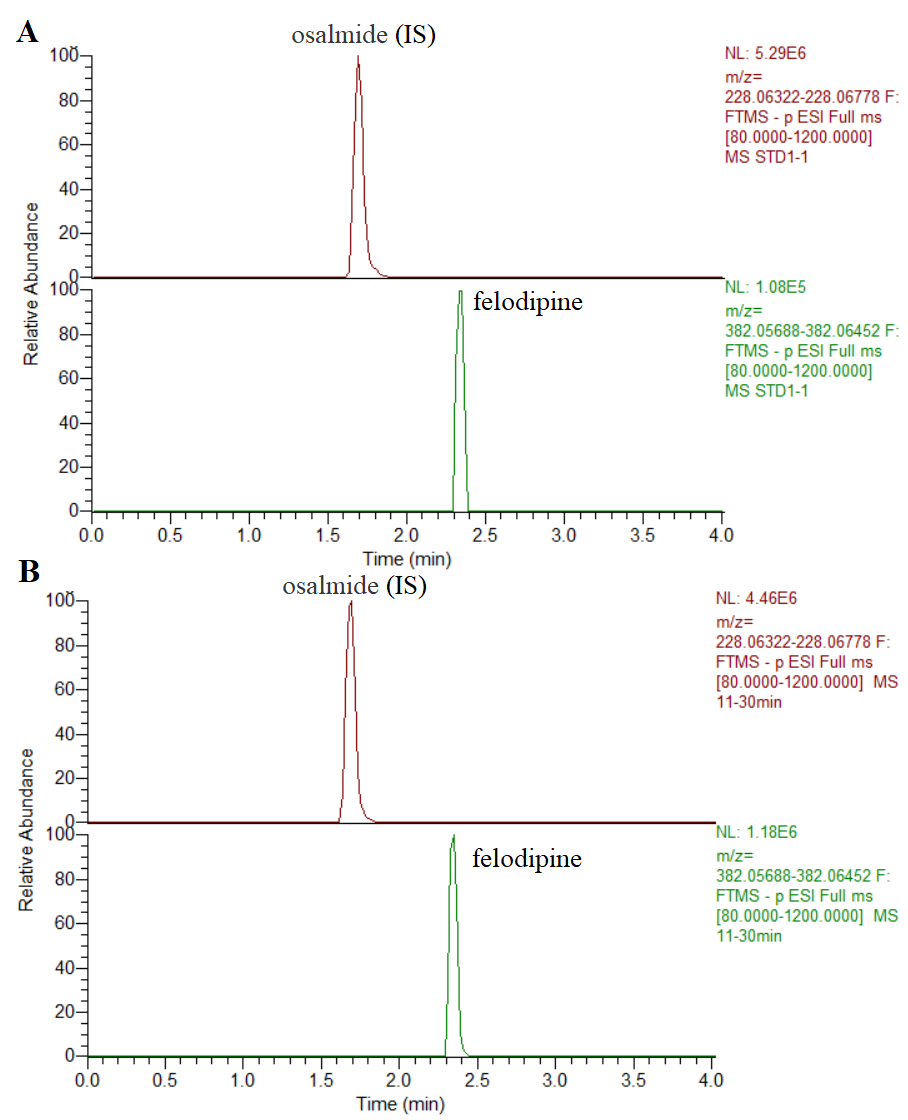


**Figure S3. UHPLC-Q-Orbitrap HRMS chromatograms of felodipine and osalmide (internal standard, IS). (**A) A blank plasma spiked with the analytes (with LLOQ) and IS (150.00 ng/mL). (B) A plasma sample (30 min) from a rat after oral administration of 10 mg/kg felodipine.


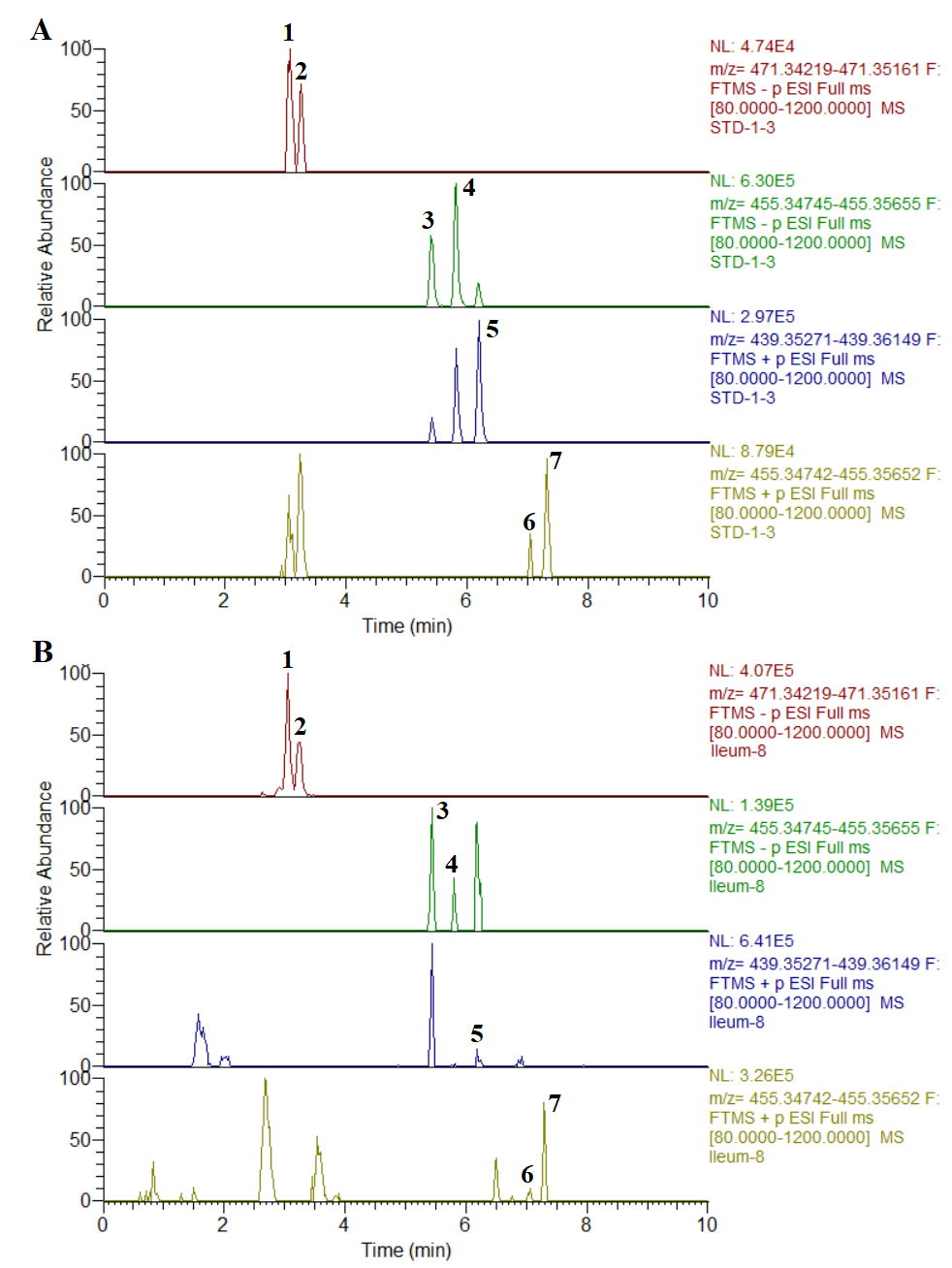


**Figure S4. UHPLC-Q-Orbitrap HRMS chromatograms of pentacyclic triterpenoid acids in Styrax. (**A) mixed reference standard reference solution (LLOQ); (B) tissue homogenate of ileum (4 h) after oral administration of 100 mg/kg Styrax. 1, maslinic acid; 2, corosolic acid; 3, epibetulinic acid; 4, betulinic acid; 5, oleanolic acid; 6, betulonic acid; 7, oleanonic acid.


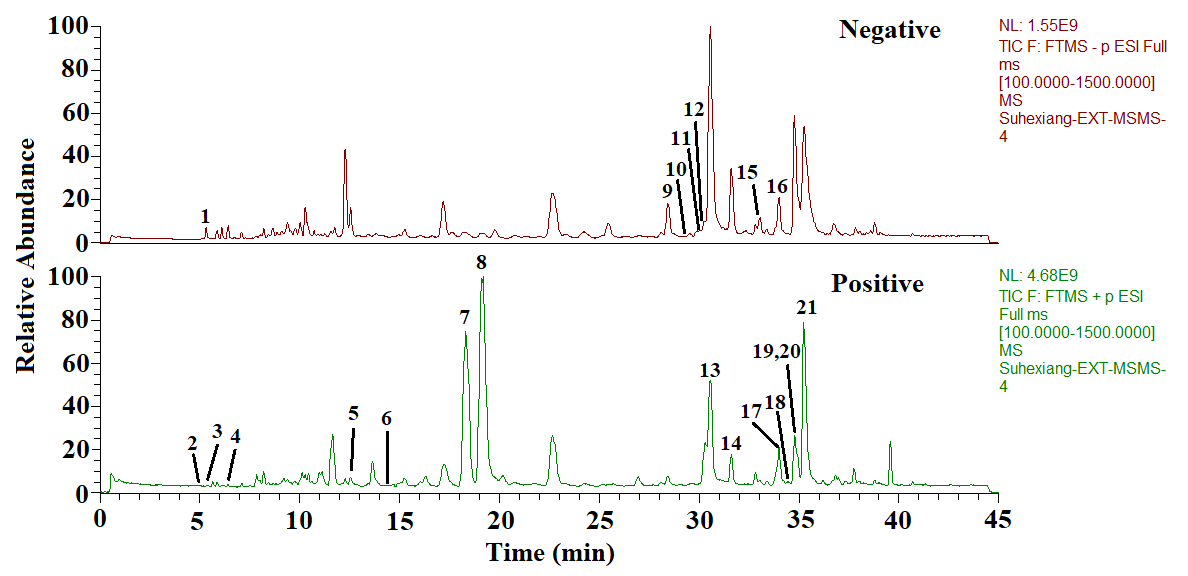


**Figure S5. The total ion chromatograms (TICs) of Styrax by UHPLC-Q-Exactive Orbitrap HRMS.**





**Figure S6.** The inhibitory effects of corosolic acid against midazolam 1’-hydroxylation in HLMs. Data are expressed as mean ± SD.





**Figure S7.** The inhibitory effects of corosolic acid against NEN-hydroxylation in HLMs. Data are expressed as mean ± SD.





**Figure S8.** Tissue distribution of corosolic acid after a single oral dose of Styrax (100 mg/kg) to rats.
